# Multi-Strategy Improved Sand Cat Swarm Optimization: Global Optimization and Feature Selection

**DOI:** 10.3390/biomimetics8060492

**Published:** 2023-10-18

**Authors:** Liguo Yao, Jun Yang, Panliang Yuan, Guanghui Li, Yao Lu, Taihua Zhang

**Affiliations:** 1 School of Mechanical and Electrical Engineering, Guizhou Normal University, Guiyang 550025, China; lgyao@gznu.edu.cn (L.Y.); juny@gznu.edu.cn (J.Y.); ghli@gznu.edu.cn (G.L.); yao.lu@gznu.edu.cn (Y.L.); 2 Technical Engineering Center of Manufacturing Service and Knowledge Engineering, Guizhou Normal University, Guiyang 550025, China; 3 State Key Laboratory of Public Big Data, Guizhou University, Guiyang 550025, China; yuanpanl2020@163.com

**Keywords:** sand cat swarm optimization, biomimetic swarm intelligence, opposition-based learning, biological elimination update mechanism, metaheuristics, benchmark, exploration and exploitation

## Abstract

The sand cat is a creature suitable for living in the desert. Sand cat swarm optimization (SCSO) is a biomimetic swarm intelligence algorithm, which inspired by the lifestyle of the sand cat. Although the SCSO has achieved good optimization results, it still has drawbacks, such as being prone to falling into local optima, low search efficiency, and limited optimization accuracy due to limitations in some innate biological conditions. To address the corresponding shortcomings, this paper proposes three improved strategies: a novel opposition-based learning strategy, a novel exploration mechanism, and a biological elimination update mechanism. Based on the original SCSO, a multi-strategy improved sand cat swarm optimization (MSCSO) is proposed. To verify the effectiveness of the proposed algorithm, the MSCSO algorithm is applied to two types of problems: global optimization and feature selection. The global optimization includes twenty non-fixed dimensional functions (Dim = 30, 100, and 500) and ten fixed dimensional functions, while feature selection comprises 24 datasets. By analyzing and comparing the mathematical and statistical results from multiple perspectives with several state-of-the-art (SOTA) algorithms, the results show that the proposed MSCSO algorithm has good optimization ability and can adapt to a wide range of optimization problems.

## 1. Introduction

With the development of the information age, there is an explosive increase in data volume. The problems people encounter in fields such as engineering [1], ecology [2], information [3], manufacturing [4], design [5], and management [6] are becoming increasingly complex. Most of these problems exhibit characteristics such as multi-objective [7] and high-dimensional [8]. The swarm intelligence algorithm is a critical way to solve optimization problems [9], with simple principles, easy implementation, and excellent performance; it has been favored by more and more scholars, and research in this area is also increasing [10]. Heuristic intelligence algorithms can achieve higher global optima by using a random search. Due to its independence in utilizing function gradients, heuristic algorithms do not require the objective function to have continuously differentiable conditions, providing optimization possibilities for some objective functions that cannot be optimized through a gradient descent [11,12].

The SCSO is a recently proposed efficient swarm intelligence algorithm that simulates the lifestyle habits of sand cats for optimization. It belongs to the evolutionary algorithm that simulates biological practices [13]. The SCSO algorithm has a simple structure and is easy to implement. Compared with the cat swarm optimization (CSO) [14], grey wolf Optimizer (GWO) [15], whale optimization algorithm (WOA) [16], salp swarm algorithm (SSA) [17], gravitational search algorithm (GSA) [18], particle swarm optimization (PSO) [19], black widow optimization (BWO) [20] and other algorithms, it has local solid development capabilities. Although the SCSO algorithm has achieved good results, due to a low population diversity and too-single exploration angle, the algorithm has a slow convergence speed and low solving accuracy, and is prone to falling into local optima during the exploration stage of complex problems. Due to the abundance of natural organisms and their easy-to-understand and accept survival habits, evolutionary algorithms that simulate biological patterns have become a hot research topic for relevant experts and scholars, such as the Genghis Khan shark optimizer (GKSO) [21], Harris hawks optimization (HHO) [22], snake optimizer (SO) [23], dung beetle optimizer (DBO) [24], crayfish optimization algorithm (COA) [25], and so on. The food chain is the foundation for the survival of the fittest, and many organisms have related shortcomings, which leads to evolutionary algorithms that simulate biological habits. Although they can solve numerous optimization problems, the optimization effect sometimes could be better. Therefore, excellent mathematical models can be obtained for optimization problem-solving after constructing a mathematical model for optimizing biological habits and improving some mathematical theories [26]. This method often achieves good optimization results. Farzad Kiani et al. proposed chaotic sand cat swarm optimization [27], Seyyedabbasi proposed binary sand cat swarm optimization algorithm [28], Amjad Qtaish et al. proposed memory-based sand cat swarm optimization [29], Wu et al. proposed modified sand cat swarm optimization algorithm [30], and Farzad Kiani et al. proposed enhanced sand cat swarm optimization inspired by the political system [31]. Our research team is also committed to improving the effectiveness of original biological intelligence algorithms by introducing some mathematical theories. We proposed algorithms such as the enhanced snake optimizer (ESO) [32], hybrid golden jackal optimization and golden sine algorithm with dynamic lens-imaging learning (LSGJO) [33], reptile search algorithm considering different flight heights (FRSA) [34], etc., to provide some ideas for solving optimization problems.

Although SCSO achieved specific results in optimization problems, it is not perfect. When encountering higher dimensional and multi-feature issues, the convergence speed of the SCSO algorithm is slow, and the disadvantage of quickly falling into local optima is fully exposed. To improve the effectiveness of the SCSO and help it overcome some physiological limitations, this paper proposes three novel strategies: a novel opposition-based learning strategy, a novel exploration mechanism, and a biological elimination update mechanism. These strategies help the SCSO quickly jump out of local optima, accelerate convergence speed, and improve optimization accuracy.

To verify the effectiveness of the MSCSO algorithm proposed in this paper, the algorithm was applied to two kinds of problems, global optimization (containing 30 functions) [35] and feature selection (containing 20 datasets) [36], which are also common complexity problems in many fields. Global optimization includes unimodal multidimensional functions, multimodal multidimensional functions, and multimodal fixed-dimensional functions. Unimodal functions are used to test the development ability of optimization algorithms, multimodal functions are used to test the exploration ability of optimization algorithms, and multidimensional functions are used to test the stability of algorithms. Feature Selection is considered an NP-hard problem: when a dataset has *N* features, 2^N^ feature subsets are generated. Metaheuristic algorithms are widely used to find an optimal solution to NP-hard problems. Such as Ma et al. [37], Wu et al. [38] and Fan et al. [39] used global optimization functions to test their proposed algorithms. Wang et al. [40], Lahmar et al. [41], and Turkoglu et al. [42] used feature selection datasets to test their proposed algorithms. Finally, by comparing the results with many advanced algorithms in global optimization and feature selection problems, it is proven that the improved algorithm proposed in this paper has excellent performance.

The main contribution of this paper is as follows:

♦ Three improvement strategies (novel opposition-based learning strategy, novel exploration mechanism, and biological elimination update mechanism) are used to improve the optimization performance of the SCSO algorithm.
♦ Thirty standard test functions for intelligent optimization algorithm testing are used to evaluate the proposed MSCSO and compare the results with 11 other advanced optimization algorithms.
♦ Twenty-four feature selection datasets were used to evaluate the proposed MSCSO and compare the results with other advanced optimization algorithms.

The chapters of this paper are arranged as follows: Section 1 introduces the background of intelligent optimization algorithms, Section 2 introduces the original SCSO, Section 3 introduces the relevant improvement strategies designed in this paper, and introduces the proposed MSCSO. Section 4 applies the proposed MSCSO to global optimization and feature selection problems and describes the corresponding statistical results. Section 5 summarizes the entire paper and looks forward to future research directions.

## 2. Original SCSO

The sand cat is the only type of cat that lives in the desert and can walk on soft, hot sand. With its superb auditory ability, it can detect low-frequency noise, detect and track prey, whether moving on the ground or underground, and then carry out capture operations on the prey. The SCSO is a novel biomimetic optimization algorithm that simulates the behavior of the sand cat in nature to achieve the optimization process. In the SCSO process, the detection and tracking of prey by sand cats can be observed as the exploration phase of the algorithm, while the capture of prey by sand cats is the exploitation phase of the algorithm.

In SCSO, the generation method of the initial solutions for the sand cat is shown in Equations (1) and (2).

(1) $$SC_{i}=[p_{i1},p_{i2},\cdots ,p_{ij},\cdots ,p_{id}]$$

(2) $$p_{ij}=ub_{j}+r_{1}\cdot (ub_{j}-lb_{j})$$

where $SC_{i}$ represents the position of the *i*-th sand cat, i∈[1,n], n is the number of populations, d is the dimension for solving the problem, $p_{ij}$ indicates the position of the *i*-th sand cat in the *j*-th dimension, $r_{1}$ is a random number between 0 and 1, $ub_{j}$ and $lb_{j}$ are the upper and lower boundary of the *j*-th dimensional space, respectively.

Due to its unique ear canal structure, sand cats can perceive low-frequency noise, which can help them make judgments based on the noise situation, search, or track prey, and achieve conversion between the stages of surround (exploration) and hunting (exploitation). R is a mathematical model for sand cats to sense low-frequency noise, as shown in Equations (3)–(5).

(3) $$r_{G}=s_{M}-(\frac{2\times s_{M}\times iter_{c}}{iter_{Max}})$$

(4) $$R=2\times r_{G}\times r_{1}-r_{G}$$

(5) $$r=r_{G}\times r_{2}$$

where $S_{M}$ is the constant 2, $r_{G}$ is a linear line that converges from 2 to 0, $iter_{c}$ represents the current number of iterations, $iter_{Max}$ represents the maximum number of iterations, R represents the transition control between exploration and exploitation, R∈[−1, 1], $r_{1}$ and $r_{2}$ are random numbers between 0 and 1, and r represents the sensitivity range of each sand cat.

When |R| > 1, The SCSO has entered the exploration phase, and its position update method is shown in Equation (6).

(6) $$P_{i,j}^{t+1}=r\times (P_{r,j}^{t}-r_{3}\times P_{i,j}^{t})$$

where $P_{i,j}^{t}$ represents the position of the *i*-th sand cat in the *j*-th dimension during the *t*-th iteration. $P_{i,j}^{t+1}$ represents the position of the *i*-th sand cat in the *j*-th dimension during the (*t+1*)-th iteration. $P_{r,j}^{t}$ is the position of the *r*-th sand cat in the *j*-th dimension of the sand cat group during the *t*-th iteration. $r_{3}$ is a random number between 0 and 1.

When |R| ≤ 1, the SCSO has entered the exploitation phase, and its location update method is shown in Equations (7) and (8).

(7) $$Pr_{i,j}^{t}=r_{4}\cdot Pb_{j}^{t}-P_{i,j}^{t}$$

(8) $$P_{i,j}^{t+1}=Pb_{j}^{t}-r\cdot Pr_{i,j}^{t}\cdot \cos(\theta )$$

where $Pr_{i,j}^{t}$ represents the random position at *t*-th iteration, which ensures that the sand cat can approach its prey. $Pb_{j}^{t}$ is the position of the optimal individual in the sand cat group in the *j*-th dimension during the *t*-th iteration. $r_{4}$ is a random number between 0 and 1. θ is assigned by the roulette wheel algorithm. $P_{i,j}^{t+1}$ represents the position of the *i*-th sand cat in the *j*-th dimension during the (*t +* 1)-th iteration.

The pseudocode of the SCSO is shown in Algorithm 1.
**Algorithm 1** The pseudocode of the SCSO1.Initialize the population Si2.Calculate the fitness function based on the objective function3.Initialize the $r,r_{G},R$ 4.**While** (t < = $iter_{Max}$)5.  **For** each search agent6.    Obtain a random angle based on the Roulette Wheel Selection (0°≤θ≤360)7.    **If** (abs(R) > 1)8.      Update the search agent position based on the Equation (6)9.    Else10.      Update the search agent position based on the Equation (8)11    **End**12.  **End**13. t = t + 114.**End**

## 3. Proposed MSCSO

The SCSO was proposed by Seyyedabbasi and Kiani in 2022 as a biomimetic intelligent algorithm. Due to sand cats’ intense hunting and survival abilities, the SCSO has excellent optimization ability by simulating their habits. But there is no free lunch in the world, and there is no one way to solve all problems [43]. When solving optimization problems, the SCSO encountered some problems. This paper introduces novel mathematical theories to improve the SCSO, enhance its effectiveness, and help it overcome some physiological limitations. This paper proposes three novel strategies, namely, a novel opposition-based learning strategy, a novel exploration mechanism, and a biological elimination update mechanism, to help the SCSO quickly jump out of local optima, accelerate convergence speed, and improve optimization accuracy.

### 3.1. Nonlinear Lens Imaging Strategy

**Figure 1:**
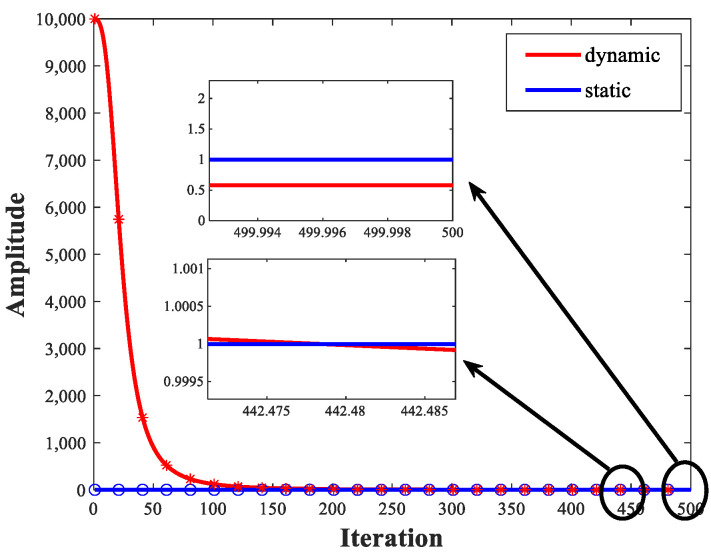
Variations of the static lens imaging strategy and the dynamic lens imaging strategy.

Lens imaging strategy is a form of opposition-based learning [44]. By refracting the object on one side of the convex lens from the convex lens to the other side of the convex lens, a more optimal solution can be obtained. However, in traditional convex lens imaging mechanisms, the imaging coefficients are often a fixed value, which is not conducive to generating population diversity. Therefore, this paper proposes a novel lens imaging strategy that expands the diversity of the population and increases the possibility of obtaining high-quality solutions by setting dynamically updated imaging coefficients. The dynamically updated imaging coefficient is defined here as ∂, which can be calculated by Equations (9)–(11). Figure 1 shows the variations of the static lens imaging strategy and the emotional lens imaging strategy. The dynamic lens imaging strategy can search for more effective regions to improve the population’s diversity and enhance the algorithm’s global search ability.

(9) $$\frac{\frac{ub+lb}{2}-P}{P^{\prime }-\frac{ub+lb}{2}}=\frac{h}{h^{\prime }}$$

(10) $$P^{\prime }=\frac{ub+lb}{2}+\frac{ub+lb}{2\times \partial }-\frac{P}{\partial }$$

(11) $$\partial =\exp({\left(iter_{c}/iter_{Max}\right)}^{3}+0.0001)-1$$

where P is the original solution; $P^{\prime }$ is a new solution obtained through the lens imaging strategy.

### 3.2. Novel Exploration Mechanisms

In the development phase of the original SCSO, assuming that the sensitivity range of the sand cat is a circle, the direction of movement can be determined by a random angle on the circle *θ*. Due to the selected arbitrary angle being between 0 and 360, its value will be between −1 and 1. In this way, each group member can move in different circumferential directions in the search space. SCSO uses a roulette selection algorithm to select a random angle for each sand cat.

**Figure 2:**
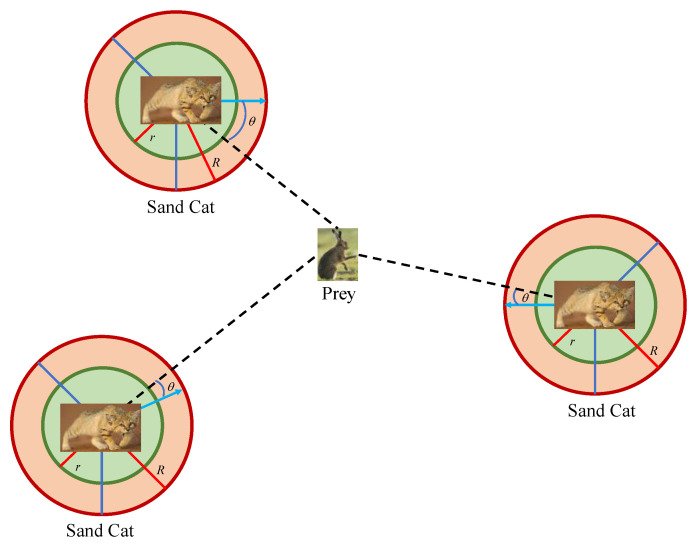
The variation form of random angle *θ*.

Inspired by this idea, this paper proposes a novel exploration mechanism by adding a random angle *θ*, enabling the sand cat to search for prey in different directions during the exploration phase. The novel exploration mechanism is represented by Equation (12). By increasing the random angle, the sand cat can approach its prey, increasing the randomness of exploration and utilizing the sand cat to close the optimal individual position. Figure 2 shows the variation form of random angle *θ*. Using this method, the sand cat can approach the hunting position while avoiding the risk of getting trapped in local optima by introducing an unexpected angle.

(12) $$P_{i,j}^{t+1}=P_{i,m_{j}}^{t}+(P_{i,m_{1}}^{t}-P_{i,m_{j}}^{t})\cdot r_{5}\cdot \cos(\theta )$$

where $P_{i,j}^{t+1}$ represents the position of the *i*-th sand cat in the *j*-th dimension during the (t+1)-th iteration. $r_{5}$ is a random number between 1 and 2. $m_{j}$ is a random number between 1 and d. $P_{i,m_{j}}^{t}$ represents the position of the *i*-th sand cat in the $m_{j}$ dimension during the *t*-th iteration. $P_{i,m_{1}}^{t}$ represents the position of the *i*-th sand cat in the $m_{1}$ dimension during the *t*-th iteration.

### 3.3. Elimination and Update Mechanism

Although the sand cat is a highly viable organism, the number of sand cat species has also changed during the exploration and exploitation stages due to changes in the external environment. Some sand cats may even be attacked by higher-level food chain species and die out. Inspired by this phenomenon, this paper proposes an elimination and update mechanism to ensure that the population size of sand cats remains consistent during the optimization process. This mechanism randomly selects 10% of individuals for elimination. If the fitness value of the new individual is lower, the old individual will be replaced, which is more in line with the survival of the fittest in the competition process of organisms. The update mechanism is shown in Equation (13).

(13) $$P{\mathrm{new}}_{i,j}^{t+1}=r_{6}\cdot r_{7}\cdot P_{i,j}^{t+1}+r_{8}\cdot (ub-lb)\sqrt{\left(\frac{iter_{Max}-iter_{c}}{iter_{Max}}\right)}$$

where, $r_{6},r_{7}$ and $r_{8}$ are random numbers between 0 and 1, respectively.

Apply the proposed improvement strategies to the SCSO and proposed the MSCSO algorithm. The pseudocode of MSCSO is shown in Algorithm 2.
**Algorithm 2.** The pseudocode of MSCSO.1.Initialize the population2.Calculate the fitness function based on the objective function3.Initialize the $r,r_{G},R$ 4.**While** (t <= $iter_{Max}$)5.  **For** each search agent6.    Obtain a random angle based on the Roulette Wheel Selection (0°≤θ≤360)7    Obtain new position by Equation (10)8    Calculate the fitness function values to obtain the optimal position9.    **If** (abs(R) > 1)10.      Update the search agent position based on the Equation (12)11.    Else12.      Update the search agent position based on the Equation (8)13    **End**14    Update new position using Equation (13)15    Check the boundaries of new position and calculate fitness value16.  **End**17  Find the current best solution18. t = t + 119.**End**

### 3.4. Time Complexity of MSCSO

In the process of optimizing practical problems, in addition to pursuing accuracy, time is also a very significant factor. The time complexity of an algorithm is an essential indicator for measuring the algorithm [45]. Therefore, it is crucial to analyze the time complexity of the improved algorithm compared to the original algorithm. The time complexity is mainly reflected in three parts: algorithm initialization, fitness evaluation, and update solution.

Time complexity is an essential indicator in algorithm comparison, representing the degree of time an algorithm takes to perform calculations, which is mainly reflected in the algorithm’s initialization, fitness evaluation, and update solution [46]. The computational complexity of SCSO is O(N×D×T), where N is the population size, D is the computational dimension of the problem, and T is the number of iterations. MSCSO has three more parts than SCSO. The time complexity of the new opposition-based learning strategy is O(D×T), and the novel exploration mechanism replaces the original exploration mechanism; thus, there is no increase in time complexity. The time complexity of the elimination update mechanism is O(0.1×N×D×T). Therefore, the time complexity of MSCSO is O(N×D×T)+O(D×T)+O(0.1×N×D×T)=O((1.1N+1)×D×T); thus, the MSCSO proposed in this paper has equal time complexity compared to SCSO.

### 3.5. Population Diversity of MSCSO

**Figure 3:**
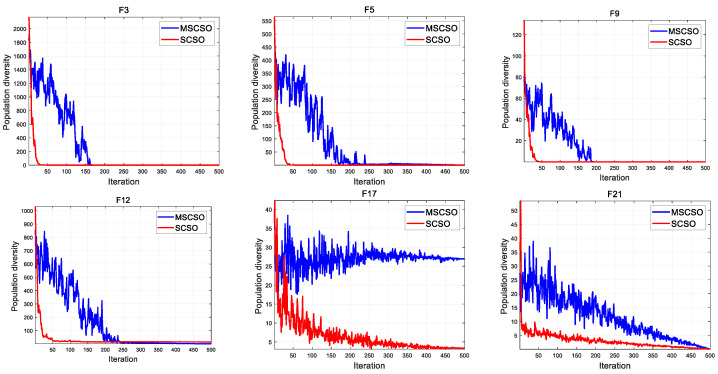
The population diversity curves of SCSO and MSCSO.

Population diversity is an important part of the qualitative analysis of algorithms. This article demonstrates the population changes of SCSO and MSCSO algorithms during optimization through population diversity experiments. Taking global optimization as an example, unimodal and multimodal functions were selected, with dimensions of 30 and fixed. The population diversity $I_{C}$ can be calculated by Equations (14) and (15) [47]. The population diversity curves of SCSO and MSCSO are shown in Figure 3.

(14) $$I_{C}(t)=\sqrt{\sum_{i=1}^{N}\sum_{d=1}^{D}{\left(x_{id}(t)-c_{d}(t)\right)}^{2}}$$

(15) $$c_{d}(t)=\frac{1}{D}\sum_{i=1}^{N}x_{id}(t)$$

where $c_{d}$ denotes the degree of dispersion between the population and the centroid c in each iteration, and $x_{id}$ represents the *d*-th dimension value of the *i*-th individual during the *t*-th iteration.

During the entire algorithm optimization process, MSCSO has higher population diversity values and better population diversity than SCSO. This indicates that MSCSO has better search ability during the exploration phase, which can avoid the algorithm falling into local optima and premature convergence.

### 3.6. Exploration and Exploitation of MSCSO

**Figure 4:**
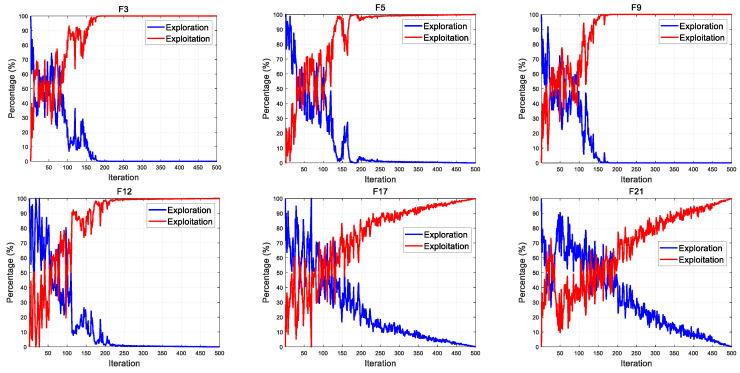
The exploration and exploitation of MSCSO.

During the optimization process, different algorithms have different design ideas, resulting in differences in exploration and exploitation. Therefore, when designing a new algorithm, it is necessary to measure the exploration and exploitation of the algorithm in order to conduct a practical analysis of the search strategies that affect these two factors. The percentage of exploration and exploitation can be calculated by Equations (16)–(18) [47]. The exploration and exploitation of MSCSO is shown in Figure 4.

(16) $$Exploration(\%)=\frac{Div(t)}{Div_{\max}}\times 100$$

(17) $$Exploitation(\%)=\frac{\left|Div(t)-Div_{\max}\right|}{Div_{\max}}\times 100$$

(18) $$Div(t)=\frac{1}{D}\sum_{d=1}^{D}\frac{1}{N}\sum_{i=1}^{N}\left|median(x_{d}(t))-x_{id}(t)\right|$$

where $Div_{\left(t\right)}$ denotes the dimension-wise diversity measurement, and $Div_{\max}$ is the maximum diversity in the whole iteration process.

During the entire algorithm optimization process, the first part of MSCSO has a high search proportion, indicating that MSCSO has good searchability, and preventing the algorithm from falling into local optima and premature convergence. In the latter part, the development proportion gradually increases, and, based on the previous search, the convergence is accelerated to obtain the optimization results. Throughout the entire optimization process, the exploration and exploitation of MSCSO maintain a dynamic balance, indicating that the algorithm has good stability and optimization performance.

## 4. Experiments and Results Analysis

### 4.1. Benchmark Datasets

In this section, we conduct performance and effectiveness testing experiments on the proposed algorithm. Global optimization and feature selection, as common problems in daily life, have become the leading choices for testing optimization algorithms used to evaluate the comprehensive ability of algorithm exploration and exploitation. In terms of global optimization, this paper selected 30 well-known functions commonly used for optimization testing as the test set, including 20 non-fixed dimensional functions and ten fixed dimensional functions. In terms of feature selection, this paper selected 24 datasets commonly used for testing. The details of the global optimization function set are shown in Appendix A Table A1. The details of datasets for feature selection are shown in Table 1, which can be obtained from the website: https://archive.ics.uci.edu/datasets (accessed on 15 October 2023).

### 4.2. Parameter Settings

In order to better compare the results with other algorithms, in the global optimization section, this paper uses 11 famous algorithms as benchmark algorithms, including GA [48], PSO [19], GWO [15], HHO [22], ACO [49], WOA [16], SOGWO [50], EGWO [51], TACPSO [52], SCSO [13], etc. These algorithms have been used as comparative methods in many studies and have excellent performance in global optimization. The details of parameter setting for algorithms are shown in Table 2.

The feature selection problem is a binary optimization problem. When applying traditional optimization algorithms to the feature selection problem, binary transformation is required first, and a transfer function is used to map continuous values to their corresponding binary values [53].

Any optimization problem is transformed into a solution for the objective function [54]. In feature selection, the goal is to minimize the number of selected features and achieve the highest accuracy. Therefore, the objective function of the feature selection problem is shown in Equation (19).

(19) $$fitness=\alpha Error+(1-\alpha )\frac{\left|S\right|}{\left|F\right|}$$

where Error represents the classification error rate, |S| represents the number of selected features, |F| represents the total number of features, and α is the weight assigned to the classification error rate, α∈[0,1].

Table 3 shows eight binary transfer functions (including four S-shaped and four V-shaped transfer functions). This paper conducted extensive simulations to verify the efficiency of these transfer functions and found that V4 is the most feasible transfer function.

In the global optimization experiment, all algorithms adopt unified parameter settings to ensure the fairness of the results. The algorithm runs independently and continuously 30 times, with a population of 50 and algorithm iterations of 500. The simulation testing environment for this time is operating system Win10, 64-bit, CPU 11th Gen Intel (R) Core (TM) i7-11700K, memory 64 GB, primary frequency 3.60 GHz, and simulation software MATLAB 2016b.

### 4.3. Results Analysis

In global optimization problems, the higher the dimension of the optimization problem, the better it can demonstrate the robustness and performance of the algorithm. Therefore, this paper focuses on 20 non-fixed dimensional functions, using three dimensions of 30, 100, and 500, respectively, to fully validate the proposed and benchmarked algorithm’s effectiveness. This paper adopts five statistical metrics to assess the effectiveness of all algorithms, including Mean, standard deviation (Std), *p*-value, Wilcoxon rank sum test, and Friedman test. It draws the iterative convergence curve and box diagram of the algorithm fully and comprehensively.

**Figure 5:**
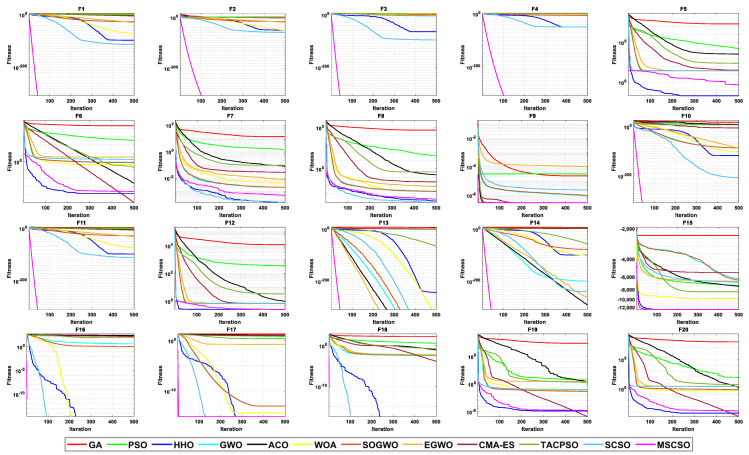
The convergence curves of 30-dimensional non-fixed dimensional functions.

**Figure 6:**
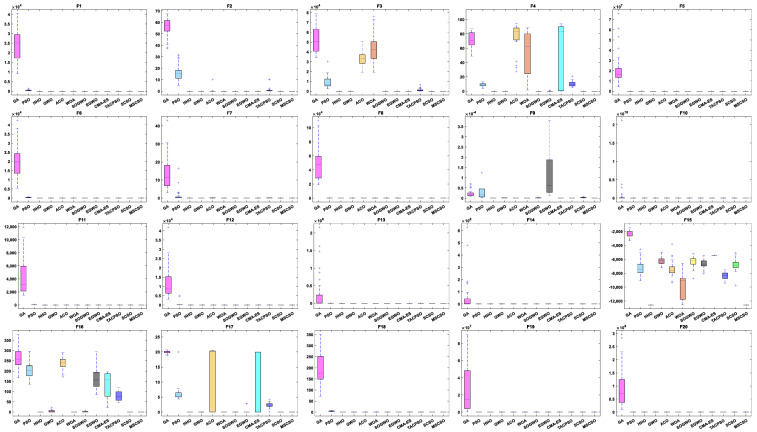
Boxplot analysis of 30-dim non-fixed dimensional functions.

Table 4 shows the results of 5 statistical metrics for 12 optimization algorithms in solving 30-dimensional non-fixed dimensional functions. Figure 5 shows the iteration curves of the 12 optimization algorithms in solving 30-dimensional non-fixed dimensional functions. Through the convergence curve, the MSCSO algorithm proposed in this paper outperforms other algorithms in terms of the convergence speed and optimization accuracy in F1, F2, F3, F4, F9, F10, F11, F13, F14, F15, F16, F17, and F18 functions. Figure 6 is a box diagram of the results of 12 optimization algorithms solving 30-dimensional non-fixed dimensional functions.

By analyzing the results in Table 4, Figure 5 and Figure 6, MSCSO achieved the most optimal values in the 30-dimensional non-fixed dimensional functions compared to the other 11 algorithms, with a quantity of 13. The Wilcoxon rank sum test and Friedman test show the overall results of each algorithm. In the Wilcoxon rank sum test, MSCSO achieved results of 190/22/8; in the Friedman test, MSCSO achieved the highest-ranking result with a value of 2.3750. The above results indicate that MSCSO has achieved better results than other algorithms in 30-dimensional non-fixed dimensional functions.

**Figure 7:**
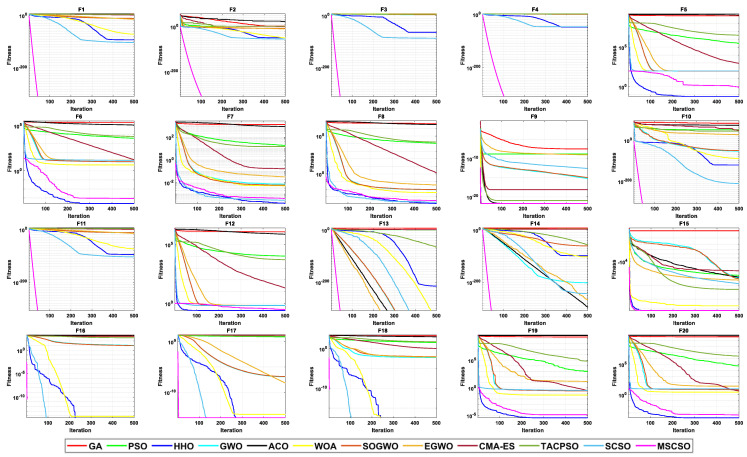
The convergence curves of 100-dimensional non-fixed dimensional functions.

**Figure 8:**
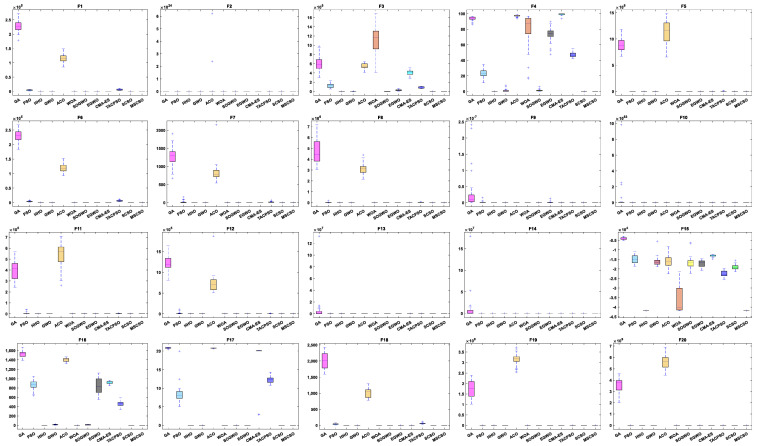
Boxplot analysis of 100-dimensional non-fixed dimensional functions.

Table 5 shows the results of 5 statistical metrics for 12 optimization algorithms in solving 100-dimensional non-fixed dimensional functions. Figure 7 shows the iteration curves of the 12 optimization algorithms in solving 100-dimensional non-fixed dimensional functions. Through the convergence curve, the MSCSO algorithm proposed in this paper outperforms other algorithms in terms of convergence speed and optimization accuracy in F1, F2, F3, F4, F9, F10, F11, F13, F14, F15, F16, F17, and F18 functions. Figure 8 is a box diagram of the results of 12 optimization algorithms solving 100-dimensional non-fixed dimensional functions.

By analyzing the results in Table 5, Figure 7 and Figure 8, MSCSO achieved the most optimal values in the 100-dimensional non-fixed dimensional functions compared to the other 11 algorithms, with a quantity of 12. The Wilcoxon rank sum test and Friedman test show the overall results of each algorithm. In the Wilcoxon rank sum test, MSCSO achieved results of 194/20/6; in the Friedman test, MSCSO achieved the highest-ranking result with a value of 2.2125. The above results indicate that MSCSO has achieved better results than other algorithms in 100-dimensional non-fixed dimensional functions.

**Figure 9:**
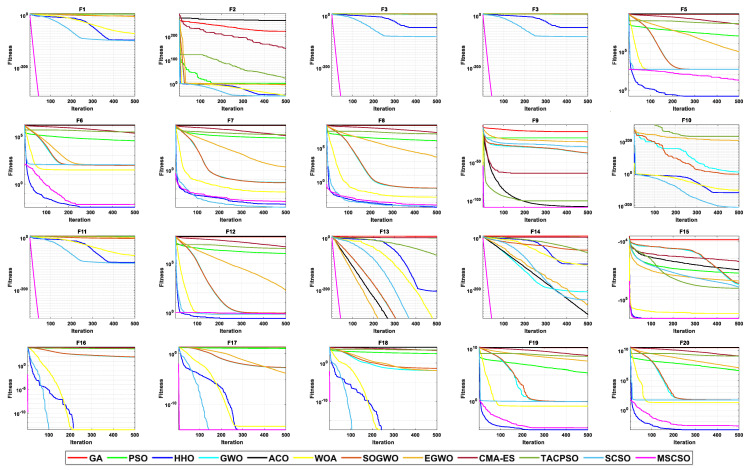
The convergence curves of 500-dimensional non-fixed dimensional functions.

**Figure 10:**
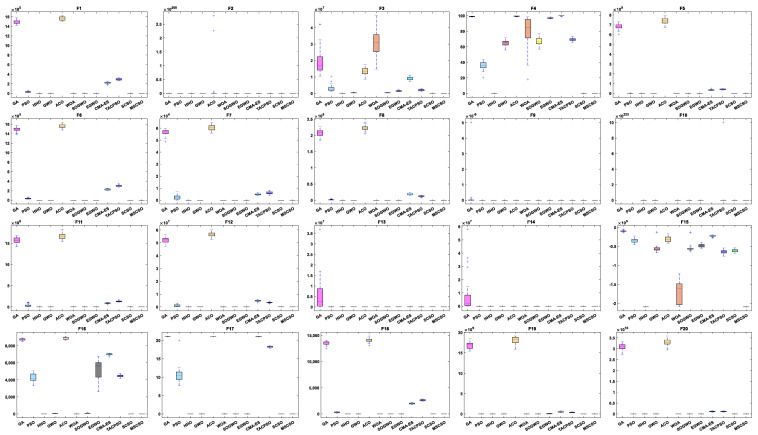
Boxplot analysis of 500-dimensional non-fixed dimensional functions.

Table 6 shows the results of 5 statistical metrics for 12 optimization algorithms in solving 500-dimensional non-fixed dimensional functions. Figure 9 shows the iteration curves of the 12 optimization algorithms in solving 500-dimensional non-fixed dimensional functions. Through the convergence curve, the MSCSO algorithm proposed in this paper outperforms other algorithms in terms of convergence speed and optimization accuracy in F1, F2, F3, F4, F9, F10, F11, F13, F14, F15, F16, F17, and F18 functions. Figure 10 is a box diagram of the results of 12 optimization algorithms solving 500-dimensional non-fixed dimensional functions.

By analyzing the results in Table 6, Figure 9 and Figure 10, MSCSO achieved the most optimal values in the 500-dimensional non-fixed dimensional functions compared to the other 11 algorithms, with a quantity of 12. The Wilcoxon rank sum test and Friedman test show the overall results of each algorithm. In the Wilcoxon rank sum test, MSCSO achieved results of 191/24/5; in the Friedman test, MSCSO achieved the highest-ranking result with a value of 2.2500. The above results indicate that MSCSO has achieved better results than other algorithms in 100-dimensional non-fixed dimensional functions.

**Figure 11:**
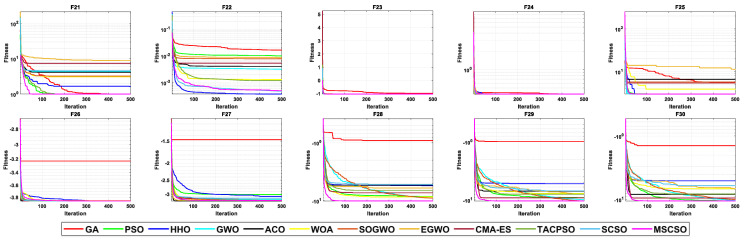
The convergence curves of fixed-dimensional functions.

**Figure 12:**
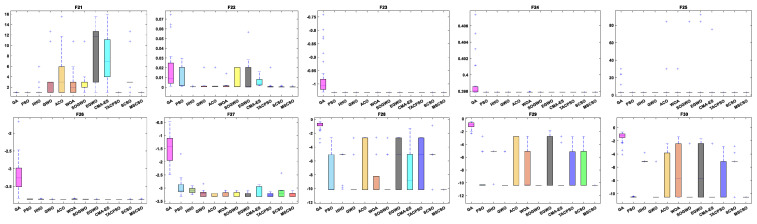
Boxplot analysis of fixed-dimensional functions.

Table 7 shows the results of 5 statistical metrics for 12 optimization algorithms in a fixeddimensional function. Figure 11 shows the iteration curves of the 12 optimization algorithms in solving fixed-dimensional functions. From the convergence curve, the MSCSO algorithm proposed in this paper has a higher convergence speed than other algorithms in functions F21, F23, F24, F28, F29, and F30. In functions F21, F23, F24, F25, F26, F28, F29, and F30, the optimization accuracy is stronger than other algorithms. Figure 12 is a box diagram of the results of 12 optimization algorithms solving fixed-dimensional functions.

Through the analysis of the results in Table 7, Figure 11 and Figure 12, in the fixed-dimensional functions, MSCSO achieved the second highest optimal value compared to the other 11 algorithms, with only one fewer number than the best TACPSO. The Wilcoxon rank sum test and Friedman test show the overall results of each algorithm. In the Wilcoxon rank sum test, MSCSO achieved a result of 60/24/26; in the Friedman test, MSCSO achieved the highest-ranking result with a value of 4.3750. The above results indicate that MSCSO has achieved better results than other algorithms in fixed-dimensional functions.

Accurate and fitness values are commonly used comparative indicators in feature selection problems. To comprehensively demonstrate the effectiveness, the average (Mean) and standard deviation (Std) of the accuracy and fitness values are calculated separately for Friedman’s test. The iterative convergence curve of the algorithm was drawn.

Table 8 shows the proposed and benchmark algorithm results for solving 24 feature selection datasets. The results include the average and standard deviation of the feature selection accuracy. In 24 datasets, MSCSO achieved 15 optimal values, with the highest number among all algorithms. Table 9 shows the Friedman test results for the average and standard deviation of feature selection accuracy, with the sum of ranks achieving 46.5 and the average of ranks achieving 1.9375, ranking first among all algorithms.

**Figure 13:**
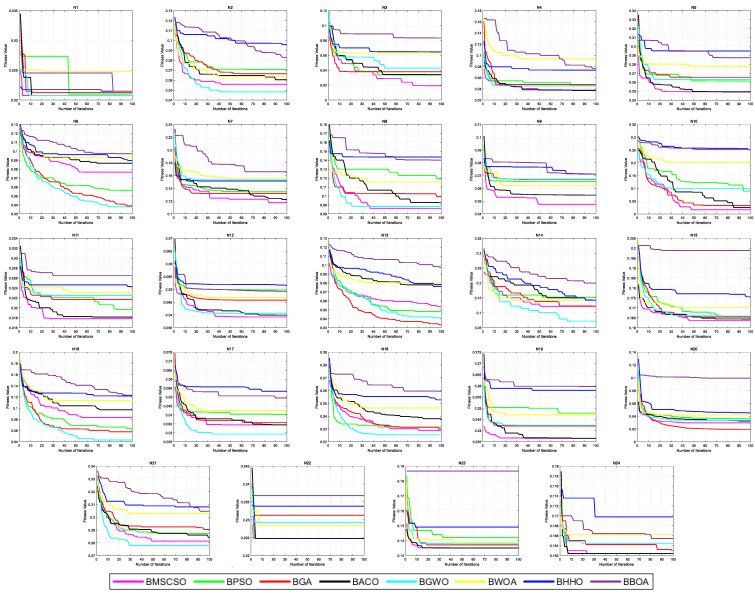
The convergence curves of feature selection datasets.

The results in Table 10 include the average and standard deviation of feature selection fitness values. In 24 datasets, MSCSO achieved 13 optimal values, with the highest number among all algorithms. Table 11 shows the Friedman test results for the average and standard deviation of feature selection accuracy, with the sum of ranks achieving 46 and the average of ranks achieving 1.9167, ranking first among all algorithms. Figure 13 shows the convergence curves of feature selection datasets.

This section validates the proposed MSCSO algorithm through two problems: global optimization and feature selection. MSCSO and other advanced algorithms were tested on 30 well-known global optimization functions (20 non-deterministic functions (Dim = 30, 100, and 500) and 10 deterministic functions) and 24 feature selection datasets. Global optimization problems such as options of five statistical metrics assess the effectiveness of all algorithms, including Mean, Std, *p*-value, Wilcoxon rank sum test, and Friedman test. The feature selection problem adopts the Mean and Std of the accuracy, and fitness values are calculated separately for Friedman’s test. The convergence curve, box diagram, and experimental data table show that MSCSO has achieved the best results, demonstrating its strong development ability and efficient spatial exploration ability. The effectiveness of the proposed strategies and methods has been verified.

## 5. Conclusions and Future Works

The SCSO is a highly effective biomimetics swarm intelligence algorithm proposed in recent years, which simulates the life habits of sand cats to optimize problems and achieve good optimization results. But in some issues, the optimization effect is not ideal. Therefore, after constructing a mathematical model for optimizing biological habits, some mathematical theories can be improved to obtain excellent optimization mathematical models. This method often achieves good optimization results. In order to enhance the effectiveness of the SCSO and help it overcome some physiological limitations, this paper proposes three novel strategies: a new opposition-based learning strategy, a new exploration mechanism, and a biological elimination update mechanism. These strategies help the SCSO easily jump out of local optima, accelerate convergence speed, and improve optimization accuracy.

To verify the effectiveness of the proposed MSCSO algorithm in this paper, the algorithm was applied to two problems: global optimization (thirty functions) and feature selection (twenty datasets). These are also common complexity problems in many fields. Finally, by comparing them with many advanced algorithms in global optimization and feature selection problems, it is proven that the improved algorithm proposed in this paper has excellent performance. Global optimization problems such as options five statistical metrics assess the effectiveness of all algorithms, including Mean, Std, *p*-value, Wilcoxon rank sum test, and Friedman test. The feature selection problem adopts the Mean and Std of the accuracy, and fitness values are calculated separately for Friedman’s test. The convergence curve, box diagram, and experimental data table show that MSCSO achieved the best results, demonstrating its strong development ability and efficient spatial exploration ability. The experimental and statistical results show that MSCSO has excellent performance and has certain advantages compared to other advanced algorithms in jumping out of local optima, improving convergence speed, and improving optimization accuracy. MSCSO has excellent optimization capabilities from both theoretical and practical perspectives. This proves that MSCSO can adapt to a wide range of optimization problems and verifies the algorithm’s robustness.

Although the strategy proposed in this article improved the optimization ability of the original SCSO, it was found through the interpretation of relevant mathematical models that the proportion of algorithm exploration and exploitation is too fixed, which cannot enable the algorithm to explore and develop according to actual problems. In later research, nonlinear dynamic adjustment factors can be set for the exploration and development stage of the algorithm. In future related research, emphasis will be placed on evolving the proposed algorithms towards more practical problems, such as feature selection in the fields of text and images. In response to the hyperparameter optimization problems faced by machine learning and deep learning, more effective heuristic algorithms will be adopted to attempt to provide some efforts for the improvement of artificial intelligence technology.

## Figures and Tables

**Table 1:** The feature selection datasets.

No.	Datasets	Features	Samples	No.	Datasets	Features	Samples
1	Iris	4	150	13	Clean1	167	476
2	Ionosphere	34	351	14	Waveform3	73	325
3	Zoo	16	101	15	PenglungEW	21	5000
4	Wine	12	178	16	Sonar	60	208
5	Glass	9	214	17	Vote	16	435
6	Musk1	467	476	18	German	24	1000
7	HeartEW	14	270	19	Diabetes	8	168
8	Lymphography	18	148	20	KrvskpEW	36	3196
9	Parkinsons	22	195	21	Australian	14	690
10	Exactly	13	1000	22	Haberman	4	306
11	Breastcancer	9	699	23	Ecoli	8	336
12	BreastEW	30	569	24	Mammographic	6	830

**Table 2:** Parameters and assignments setting for algorithms.

Algorithms	Parameters and Assignments
GA	α∈[−0.5, 1.5]
PSO	$c_{1}=2$, $c_{2}=2$, $W_{\min}=0.2$, $W_{\max}=0.9$
GWO	a=2(linearly decreases over iterations), $r_{1}\in [0,1]$, $r_{2}\in [0,1]$
HHO	J∈[0,2]
ACO	α=1, β=2, ρ=0.05
WOA	a∈[2,0], A∈[2,0],C=2.rand(0,1),l∈[−1,1],b=1
SOGWO	a=2(linearly decreases over iterations), $r_{1}\in [0,1]$, $r_{2}\in [0,1]$
EGWO	a=2(linearly decreases over iterations), $r_{1}\in [0,1]$, $r_{2}\in [0,1]$
TACPSO	$c_{1}=2$, $c_{2}=2$, $W_{\min}=0.2$, $W_{\max}=0.9$
SCSO	$r_{G}\in \left[2,0\right]$, $R\in \left[-2r_{G},2r_{G}\right]$
MSCSO	$r_{G}\in \left[2,0\right]$, $R\in \left[-2r_{G},2r_{G}\right]$

**Table 3:** Details of binary transfer functions.

S-Shaped	Transfer Functions	V-Shaped	Transfer Functions
S1	$T(x)=\frac{1}{1+e^{-2x}}$	V1	$T(x)=\left|erf(\frac{\sqrt{\pi }}{2}x)\right|$
S2	$T(x)=\frac{1}{1+e^{-2x}}$	V2	T(x)=|tanh(x)|
S3	$T(x)=\frac{1}{1+e^{\left(-x/2\right)}}$	V3	$T(x)=\left|(x)/\sqrt{1+x^{2}}\right|$
S4	$T(x)=\frac{1}{1+e^{\left(-x/3\right)}}$	V4	$T(x)=\left|\frac{2}{\pi }\arctan(\frac{\pi }{2}x)\right|$

**Table 4:** Comparison of results on 30-dimensional non-fixed dimensional functions.

F(x)	Metric	GA	PSO	GWO	HHO	ACO	WOA	CMA-ES	SOGWO	EGWO	TACPSO	SCSO	MSCSO
F1	Mean	2.3882 × 10^04^	4.0252 × 10^02^	9.4854 × 10^−28^	1.0834 × 10^−97^	3.1450 × 10^−03^	1.2385 × 10^−74^	1.0751 × 10^−05^	2.5836 × 10^−27^	1.2941 × 10^−30^	1.5302 × 10^−01^	4.6963 × 10^−114^	**0.0000 × 10^00^**
	Std	8.0237 × 10^03^	2.4914 × 10^02^	9.6209 × 10^−28^	4.7523 × 10^−97^	3.4279 × 10^−03^	2.7928 × 10^−74^	4.1260 × 10^−06^	4.2308 × 10^−27^	4.8761 × 10^−30^	3.4246 × 10^−01^	1.5788 × 10^−113^	**0.0000 × 10^00^**
	P	1.2118 × 10^−12^	1.2118 × 10^−12^	1.2118 × 10^−12^	1.2118 × 10^−12^	1.2118 × 10^−12^	1.2118 × 10^−12^	1.2118 × 10^−12^	1.2118 × 10^−12^	1.2118 × 10^−12^	1.2118 × 10^−12^	1.2118 × 10^−12^	——
	Wr	(+)	(+)	(+)	(+)	(+)	(+)	(+)	(+)	(+)	(+)	(+)	——
F2	Mean	5.5958 × 10^01^	1.6450 × 10^01^	8.0038 × 10^−17^	2.3736 × 10^−50^	6.7519 × 10^−01^	7.3908 × 10^−51^	4.8772 × 10^−03^	7.6346 × 10^−17^	1.8008 × 10^−19^	1.1305 × 10^00^	2.9535 × 10^−59^	**0.0000 × 10^00^**
	Std	8.2859 × 10^00^	7.1315 × 10^00^	5.9003 × 10^−17^	9.1308 × 10^−50^	2.5363 × 10^00^	3.2765 × 10^−50^	1.2516 × 10^−03^	5.8232 × 10^−17^	4.9257 × 10^−19^	2.5175 × 10^00^	1.4573 × 10^−58^	**0.0000 × 10^00^**
	P	1.2118 × 10^−12^	1.2118 × 10^−12^	1.2118 × 10^−12^	1.2118 × 10^−12^	1.2118 × 10^−12^	1.2118 × 10^−12^	1.2118 × 10^−12^	1.2118 × 10^−12^	1.2118 × 10^−12^	1.2118 × 10^−12^	1.2118 × 10^−12^	——
	Wr	(+)	(+)	(+)	(+)	(+)	(+)	(+)	(+)	(+)	(+)	(+)	——
F3	Mean	5.1764 × 10^04^	9.1934 × 10^03^	8.4566 × 10^−06^	2.0375 × 10^−65^	3.3823 × 10^04^	4.4908 × 10^04^	5.3514 × 10^00^	1.3368 × 10^−04^	5.5742 × 10^−04^	1.3350 × 10^03^	8.6436 × 10^−97^	**0.0000 × 10^00^**
	Std	1.3440 × 10^04^	5.9570 × 10^03^	1.9506 × 10^−05^	1.1160 × 10^−64^	7.1006 × 10^03^	1.3375 × 10^04^	1.5242 × 10^01^	3.8736 × 10^−04^	2.7963 × 10^−03^	1.3539 × 10^03^	2.6872 × 10^−96^	**0.0000 × 10^00^**
	P	1.2118 × 10^−12^	1.2118 × 10^−12^	1.2118 × 10^−12^	1.2118 × 10^−12^	1.2118 × 10^−12^	1.2118 × 10^−12^	1.2118 × 10^−12^	1.2118 × 10^−12^	1.2118 × 10^−12^	1.2118 × 10^−12^	1.2118 × 10^−12^	——
	Wr	(+)	(+)	(+)	(+)	(+)	(+)	(+)	(+)	(+)	(+)	(+)	——
F4	Mean	7.1899 × 10^01^	8.9183 × 10^00^	7.7056 × 10^−07^	6.4756 × 10^−50^	7.6759 × 10^01^	5.1504 × 10^01^	5.3379 × 10^01^	1.7477 × 10^−06^	5.9639 × 10^−02^	1.0062 × 10^01^	4.1841 × 10^−50^	**0.0000 × 10^00^**
	Std	9.7846 × 10^00^	2.4777 × 10^00^	9.2735 × 10^−07^	1.6146 × 10^−49^	1.8395 × 10^01^	2.5189 × 10^01^	4.2940 × 10^01^	3.2233 × 10^−06^	2.3079 × 10^−01^	3.8593 × 10^00^	1.4644 × 10^−49^	**0.0000 × 10^00^**
	P	1.2118 × 10^−12^	1.2118 × 10^−12^	1.2118 × 10^−12^	1.2118 × 10^−12^	1.2118 × 10^−12^	1.2118 × 10^−12^	1.2118 × 10^−12^	1.2118 × 10^−12^	1.2118 × 10^−12^	1.2118 × 10^−12^	1.2118 × 10^−12^	——
	Wr	(+)	(+)	(+)	(+)	(+)	(+)	(+)	(+)	(+)	(+)	(+)	——
F5	Mean	2.1880 × 10^07^	1.7601 × 10^04^	2.7143 × 10^01^	**1.9384 × 10^−02^**	3.4312 × 10^03^	2.7964 × 10^01^	3.1524 × 10^01^	2.7193 × 10^01^	2.8053 × 10^01^	2.3597 × 10^02^	2.8028 × 10^01^	4.4841 × 10^−01^
	Std	1.6446 × 10^07^	1.8892 × 10^04^	8.1726 × 10^−01^	**3.0642 × 10^−02^**	1.6368 × 10^04^	4.7168 × 10^−01^	2.4178 × 10^01^	7.6903 × 10^−01^	7.7618 × 10^−01^	5.3466 × 10^02^	8.7610 × 10^−01^	1.2419 × 10^00^
	P	3.0199 × 10^−11^	3.0199 × 10^−11^	3.0199 × 10^−11^	1.8608 × 10^−06^	3.0199 × 10^−11^	3.0199 × 10^−11^	3.0199 × 10^−11^	3.0199 × 10^−11^	3.0199 × 10^−11^	3.0199 × 10^−11^	3.0199 × 10^−11^	——
	Wr	(+)	(+)	(+)	(−)	(+)	(+)	(+)	(+)	(+)	(+)	(+)	——
F6	Mean	2.0342 × 10^04^	3.5598 × 10^02^	8.1840 × 10^−01^	**1.5320 × 10^−04^**	2.5508 × 10^−03^	4.1990 × 10^−01^	1.1798 × 10^−05^	7.3664 × 10^−01^	3.3872 × 10^00^	1.8392 × 10^−01^	1.8160 × 10^00^	2.5493 × 10^−04^
	Std	9.4131 × 10^03^	1.5762 × 10^02^	3.8138 × 10^−01^	**1.5441 × 10^−04^**	2.4175 × 10^−03^	2.2643 × 10^−01^	5.6766 × 10^−06^	3.8153 × 10^−01^	6.0128 × 10^−01^	3.8507 × 10^−01^	5.2389 × 10^−01^	3.2794 × 10^−04^
	P	3.0199 × 10^−11^	3.0199 × 10^−11^	3.0199 × 10^−11^	1.3732 × 10^−01^	1.1737 × 10^−09^	3.0199 × 10^−11^	6.7220 × 10^−10^	3.0199 × 10^−11^	3.0199 × 10^−11^	3.3384 × 10^−11^	3.0199 × 10^−11^	——
	Wr	(+)	(+)	(+)	(=)	(+)	(+)	(−)	(+)	(+)	(+)	(+)	——
F7	Mean	1.3664 × 10^01^	1.4687 × 10^00^	1.9841 × 10^−03^	1.4422 × 10^−04^	8.0345 × 10^−02^	3.7002 × 10^−03^	2.6953 × 10^−02^	2.0066 × 10^−03^	7.5552 × 10^−03^	9.1955 × 10^−02^	**1.3950 × 10^−04^**	5.0156 × 10^−04^
	Std	8.7006 × 10^00^	3.2863 × 10^00^	1.0485 × 10^−03^	1.5180 × 10^−04^	2.9376 × 10^−02^	4.5906 × 10^−03^	6.3070 × 10^−03^	7.3785 × 10^−04^	4.0167 × 10^−03^	4.1518 × 10^−02^	**1.4087 × 10^−04^**	6.8478 × 10^−04^
	P	3.0199 × 10^−11^	3.0199 × 10^−11^	2.1947 × 10^−08^	6.6689 × 10^−03^	3.0199 × 10^−11^	4.1127 × 10^−07^	3.0199 × 10^−11^	4.5726 × 10^−09^	4.0772 × 10^−11^	3.0199 × 10^−11^	4.8560 × 10^−03^	——
	Wr	(+)	(+)	(+)	(−)	(+)	(+)	(+)	(+)	(+)	(+)	(−)	——
F8	Mean	4.8999 × 10^04^	3.6819 × 10^01^	2.1156 × 10^−03^	1.3081 × 10^−04^	1.9022 × 10^−01^	5.5122 × 10^−03^	2.5917 × 10^−02^	1.8879 × 10^−03^	7.1894 × 10^−03^	4.3129 × 10^−01^	**8.3222 × 10^−05^**	2.2013 × 10^−04^
	Std	2.4132 × 10^04^	3.5570 × 10^01^	1.1600 × 10^−03^	1.3126 × 10^−04^	7.7122 × 10^−02^	6.3741 × 10^−03^	8.7268 × 10^−03^	1.1092 × 10^−03^	3.4171 × 10^−03^	2.3226 × 10^−01^	**1.1264 × 10^−04^**	2.2498 × 10^−04^
	P	3.0199 × 10^−11^	3.0199 × 10^−11^	5.4941 × 10^−11^	9.9258 × 10^−02^	3.0199 × 10^−11^	7.6950 × 10^−08^	3.0199 × 10^−11^	5.4941 × 10^−11^	3.0199 × 10^−11^	3.0199 × 10^−11^	4.0330 × 10^−03^	——
	Wr	(+)	(+)	(+)	(=)	(+)	(+)	(+)	(+)	(+)	(+)	(−)	——
F9	Mean	2.3492 × 10^−05^	3.2403 × 10^−05^	9.0901 × 10^−07^	**3.0590 × 10^−07^**	**3.0590 × 10^−07^**	**3.0590 × 10^−07^**	**3.0590 × 10^−07^**	9.7708 × 10^−07^	1.0893 × 10^−04^	**3.0590 × 10^−07^**	2.3376 × 10^−06^	**3.0590 × 10^−07^**
	Std	1.7190 × 10^−05^	3.9762 × 10^−05^	5.6252 × 10^−07^	**2.6922 × 10^−22^**	**2.6922 × 10^−22^**	**2.6922 × 10^−22^**	**2.6922 × 10^−22^**	7.3193 × 10^−07^	1.0355 × 10^−04^	**2.6922 × 10^−22^**	1.5292 × 10^−06^	**2.6922 × 10^−22^**
	P	1.2118 × 10^−12^	1.0239 × 10^−12^	1.2118 × 10^−12^	NaN	NaN	NaN	NaN	1.2118 × 10^−12^	1.2118 × 10^−12^	NaN	1.2118 × 10^−12^	——
	Wr	(+)	(+)	(+)	(=)	(=)	(=)	(=)	(+)	(+)	(=)	(+)	——
F10	Mean	1.0145 × 10^17^	8.4409 × 10^08^	8.1966 × 10^−87^	5.5583 × 10^−118^	3.3982 × 10^08^	7.6823 × 10^−102^	1.8948 × 10^−06^	6.0445 × 10^−87^	2.1559 × 10^−88^	3.7142 × 10^06^	2.7945 × 10^−207^	**0.0000 × 10^00^**
	Std	3.9055 × 10^17^	2.6029 × 10^09^	4.2284 × 10^−86^	2.1965 × 10^−117^	1.8250 × 10^09^	4.2059 × 10^−101^	2.9163 × 10^−06^	3.1459 × 10^−86^	1.1808 × 10^−87^	1.8277 × 10^07^	0.0000 × 10^00^	**0.0000 × 10^00^**
	P	1.2118 × 10^−12^	1.2118 × 10^−12^	1.2118 × 10^−12^	1.2118 × 10^−12^	1.2118 × 10^−12^	1.2118 × 10^−12^	1.2118 × 10^−12^	1.2118 × 10^−12^	1.2118 × 10^−12^	1.2118 × 10^−12^	1.2118 × 10^−12^	——
	Wr	(+)	(+)	(+)	(+)	(+)	(+)	(+)	(+)	(+)	(+)	(+)	——
F11	Mean	4.1073 × 10^03^	1.9695 × 10^01^	1.2612 × 10^−28^	6.3211 × 10^−98^	1.3240 × 10^−03^	2.7798 × 10^−76^	5.5566 × 10^−07^	1.3055 × 10^−28^	6.1163 × 10^−32^	2.3031 × 10^−02^	6.5380 × 10^−111^	**0.0000 × 10^00^**
	Std	2.5445 × 10^03^	9.5147 × 10^00^	1.9626 × 10^−28^	3.4427 × 10^−97^	1.9224 × 10^−03^	9.6563 × 10^−76^	2.5350 × 10^−07^	2.5006 × 10^−28^	1.4303 × 10^−31^	7.7778 × 10^−02^	3.5781 × 10^−110^	**0.0000 × 10^00^**
	P	1.2118 × 10^−12^	1.2118 × 10^−12^	1.2118 × 10^−12^	1.2118 × 10^−12^	1.2118 × 10^−12^	1.2118 × 10^−12^	1.2118 × 10^−12^	1.2118 × 10^−12^	1.2118 × 10^−12^	1.2118 × 10^−12^	1.2118 × 10^−12^	——
	Wr	(+)	(+)	(+)	(+)	(+)	(+)	(+)	(+)	(+)	(+)	(+)	——
F12	Mean	1.1708 × 10^04^	3.4868 × 10^02^	6.6669 × 10^−01^	2.4973 × 10^−01^	9.6624 × 10^−01^	6.6701 × 10^−01^	6.6667 × 10^−01^	6.6668 × 10^−01^	6.7791 × 10^−01^	3.2344 × 10^00^	6.6667 × 10^−01^	**2.4509 × 10^−01^**
	Std	8.6335 × 10^03^	1.2065 × 10^03^	5.8385 × 10^−05^	7.7056 × 10^−04^	7.7851 × 10^−01^	3.9840 × 10^−04^	1.3368 × 10^−05^	2.6822 × 10^−05^	6.0836 × 10^−02^	4.0159 × 10^00^	**2.1578 × 10^−07^**	1.1400 × 10^−02^
	P	3.0199 × 10^−11^	3.0199 × 10^−11^	3.0199 × 10^−11^	1.0407 × 10^−04^	3.0199 × 10^−11^	3.0199 × 10^−11^	3.0199 × 10^−11^	3.0199 × 10^−11^	3.0199 × 10^−11^	3.0199 × 10^−11^	3.0199 × 10^−11^	——
	Wr	(+)	(+)	(+)	(+)	(+)	(+)	(+)	(+)	(+)	(+)	(+)	——
F13	Mean	2.9962 × 10^05^	4.9379 × 10^00^	**0.0000 × 10^00^**	6.1378 × 10^−243^	**0.0000 × 10^00^**	**0.0000 × 10^00^**	2.7895 × 10^−01^	**0.0000 × 10^00^**	**0.0000 × 10^00^**	2.2045 × 10^−67^	**0.0000 × 10^00^**	**0.0000 × 10^00^**
	Std	5.6755 × 10^05^	1.4569 × 10^01^	**0.0000 × 10^00^**	**0.0000 × 10^00^**	**0.0000 × 10^00^**	**0.0000 × 10^00^**	8.4603 × 10^−01^	**0.0000 × 10^00^**	**0.0000 × 10^00^**	7.4589 × 10^−67^	**0.0000 × 10^00^**	**0.0000 × 10^00^**
	P	1.2118 × 10^−12^	1.2118 × 10^−12^	1.2118 × 10^−12^	1.7016 × 10^−08^	NaN	NaN	1.2118 × 10^−12^	NaN	NaN	1.2118 × 10^−12^	NaN	——
	Wr	(+)	(+)	(+)	(+)	(=)	(=)	(+)	(=)	(=)	(+)	(=)	——
F14	Mean	6.6487 × 10^05^	1.4240 × 10^03^	1.0865 × 10^−199^	2.1045 × 10^−100^	3.5179 × 10^−291^	7.7139 × 10^−107^	4.5316 × 10^01^	2.5201 × 10^−78^	1.5299 × 10^−262^	6.3626 × 10^−57^	1.8398 × 10^−240^	**0.0000 × 10^00^**
	Std	1.4463 × 10^06^	3.4291 × 10^03^	**0.0000 × 10^00^**	1.1527 × 10^−99^	**0.0000 × 10^00^**	2.8284 × 10^−106^	3.7111 × 10^01^	1.3803 × 10^−77^	**0.0000 × 10^00^**	2.8667 × 10^−56^	**0.0000 × 10^0^** ^0^	**0.0000 × 10^00^**
	P	1.2118 × 10^−12^	1.2118 × 10^−12^	1.2118 × 10^−12^	1.2118 × 10^−12^	1.2118 × 10^−12^	1.2118 × 10^−12^	1.2118 × 10^−12^	1.2118 × 10^−12^	5.8522 × 10^−09^	1.2118 × 10^−12^	1.2118 × 10^−12^	——
	Wr	(+)	(+)	(+)	(+)	(+)	(+)	(+)	(+)	(+)	(+)	(+)	——
F15	Mean	−2.3265 × 10^03^	−7.2536 × 10^03^	−6.2034 × 10^03^	**−1.2569 × 10^04^**	−7.3992 × 10^03^	−9.6875 × 10^03^	−5.4185 × 10^03^	−6.4035 × 10^03^	−6.6208 × 10^03^	−8.3848 × 10^03^	−6.7829 × 10^03^	−1.2568 × 10^04^
	Std	4.9200 × 10^02^	1.0589 × 10^03^	5.9953 × 10^02^	**7.9718 × 10^−01^**	1.1202 × 10^03^	1.8009 × 10^03^	2.8361 × 10^00^	7.6384 × 10^02^	6.1800 × 10^02^	5.2569 × 10^02^	8.8936 × 10^02^	1.4088 × 10^00^
	P	3.0199 × 10^−11^	3.0199 × 10^−11^	3.0199 × 10^−11^	1.0188 × 10^−05^	3.0199 × 10^−11^	3.0199 × 10^−11^	3.1602 × 10^−12^	3.1602 × 10^−12^	3.1602 × 10^−12^	3.1602 × 10^−12^	3.1602 × 10^−12^	——
	Wr	(+)	(+)	(+)	(−)	(+)	(+)	(+)	(+)	(+)	(+)	(+)	——
F16	Mean	2.6969 × 10^02^	2.0122 × 10^02^	3.9097 × 10^00^	**0.0000 × 10^00^**	2.3545 × 10^02^	1.8948 × 10^−15^	1.3219 × 10^02^	1.1182 × 10^00^	1.6074 × 10^02^	8.0006 × 10^01^	**0.0000 × 10^00^**	**0.0000 × 10^00^**
	Std	5.1650 × 10^01^	3.7574 × 10^01^	4.9670 × 10^00^	**0.0000 × 10^00^**	2.8249 × 10^01^	1.0378 × 10^−14^	6.4843 × 10^01^	1.8024 × 10^00^	4.5943 × 10^01^	2.2926 × 10^01^	**0.0000 × 10^00^**	**0.0000 × 10^00^**
	P	1.2118 × 10^−12^	1.2118 × 10^−12^	1.1921 × 10^−12^	NaN	1.2118 × 10^−12^	3.3371 × 10^−01^	1.2118 × 10^−12^	1.1462 × 10^−12^	1.2118 × 10^−12^	1.2118 × 10^−12^	NaN	——
	Wr	(+)	(+)	(+)	(=)	(+)	(+)	(+)	(+)	(+)	(+)	(=)	——
F17	Mean	1.9918 × 10^01^	6.2986 × 10^00^	1.0427 × 10^−13^	**8.8818 × 10^−16^**	1.2668 × 10^01^	4.7962 × 10^−15^	1.0646 × 10^01^	1.0451 × 10^−13^	1.8196 × 10^−01^	2.3619 × 10^00^	**8.8818 × 10^−16^**	**8.8818 × 10^−16^**
	Std	4.0001 × 10^−01^	2.7508 × 10^00^	1.5169 × 10^−14^	**0.0000 × 10^00^**	9.8240 × 10^00^	2.1580 × 10^−15^	1.0128 × 10^01^	1.8093 × 10^−14^	6.9249 × 10^−01^	9.2248 × 10^−01^	**0.0000 × 10^00^**	**0.0000 × 10^00^**
	P	1.2118 × 10^−12^	1.2118 × 10^−12^	1.0947 × 10^−12^	NaN	1.2118 × 10^−12^	8.0416 × 10^−11^	1.2118 × 10^−12^	1.1453 × 10^−12^	8.6036 × 10^−13^	1.2118 × 10^−12^	NaN	——
	Wr	(+)	(+)	(+)	(=)	(+)	(+)	(+)	(+)	(+)	(+)	(=)	——
F18	Mean	1.9614 × 10^02^	4.0914 × 10^00^	3.5675 × 10^−03^	**0.0000 × 10^00^**	8.9299 × 10^−02^	5.0132 × 10^−03^	1.3486 × 10^−04^	5.0762 × 10^−03^	5.8190 × 10^−03^	1.8384 × 10^−01^	**0.0000 × 10^00^**	**0.0000 × 10^00^**
	Std	7.7683 × 10^01^	1.6958 × 10^00^	7.6611 × 10^−03^	**0.0000 × 10^00^**	1.7726 × 10^−01^	2.7459 × 10^−02^	4.9410 × 10^−05^	9.6453 × 10^−03^	9.3203 × 10^−03^	2.0792 × 10^−01^	**0.0000 × 10^00^**	**0.0000 × 10^00^**
	P	1.2118 × 10^−12^	1.2118 × 10^−12^	1.1035 × 10^−02^	NaN	1.2118 × 10^−12^	3.3371 × 10^−01^	1.2118 × 10^−12^	2.7880 × 10^−03^	1.4552 × 10^−04^	1.2118 × 10^−12^	NaN	——
	Wr	(+)	(+)	(+)	(=)	(+)	(=)	(+)	(+)	(+)	(+)	(=)	——
F19	Mean	2.3968 × 10^07^	5.3044 × 10^00^	5.3218 × 10^−02^	1.2764 × 10^−05^	2.4202 × 10^00^	2.3134 × 10^−02^	**1.2759 × 10^−06^**	4.7523 × 10^−02^	2.7727 × 10^00^	2.0992 × 10^00^	1.0182 × 10^−01^	1.6282 × 10^−05^
	Std	2.4219 × 10^07^	2.2030 × 10^00^	2.7722 × 10^−02^	1.1330 × 10^−05^	2.8971 × 10^00^	2.7220 × 10^−02^	**4.8945 × 10^−07^**	3.3122 × 10^−02^	2.9934 × 10^00^	1.7822 × 10^00^	4.8569 × 10^−02^	1.7897 × 10^−05^
	P	3.0199 × 10^−11^	3.0199 × 10^−11^	3.0199 × 10^−11^	7.3940 × 10^−01^	3.0199 × 10^−11^	3.0199 × 10^−11^	5.8587 × 10^−06^	3.0199 × 10^−11^	3.0199 × 10^−11^	3.0199 × 10^−11^	3.0199 × 10^−11^	——
	Wr	(+)	(+)	(+)	(=)	(+)	(+)	(−)	(+)	(+)	(+)	(+)	——
F20	Mean	9.3930 × 10^07^	7.9378 × 10^01^	7.1729 × 10^−01^	6.5993 × 10^−05^	1.5778 × 10^00^	5.5696 × 10^−01^	**1.7743 × 10^−05^**	6.7547 × 10^−01^	2.5680 × 10^00^	4.8580 × 10^00^	2.4786 × 10^00^	1.4439 × 10^−04^
	Std	7.8709 × 10^07^	3.0201 × 10^02^	2.2320 × 10^−01^	9.3433 × 10^−05^	2.2858 × 10^00^	2.3534 × 10^−01^	**9.7499 × 10^−06^**	2.3352 × 10^−01^	3.9348 × 10^−01^	5.6359 × 10^00^	3.1401 × 10^−01^	2.2463 × 10^−04^
	P	3.0199 × 10^−11^	3.0199 × 10^−11^	3.0199 × 10^−11^	2.1156 × 10^−01^	3.0199 × 10^−11^	3.0199 × 10^−11^	8.5641 × 10^−04^	3.0199 × 10^−11^	3.0199 × 10^−11^	3.0199 × 10^−11^	3.0199 × 10^−11^	——
	Wr	(+)	(+)	(+)	(=)	(+)	(+)	(−)	(+)	(+)	(+)	(+)	——
Wilcoxon’s rank sum test		20/0/0	20/0/0	20/0/0	9/8/3	18/2/0	17/3/0	16/1/3	19/1/0	19/1/0	19/1/0	13/5/2	——
Friedman value		1.1450 × 10^01^	1.0350 × 10^01^	5.4625 × 10^00^	2.8875 × 10^00^	8.2375 × 10^00^	5.4625 × 10^00^	6.7625 × 10^00^	5.8125 × 10^00^	6.9125 × 10^00^	8.4750 × 10^00^	3.8125 × 10^00^	**2.3750 × 10^00^**
Friedman rank		12	11	4	2	9	4	7	6	8	10	3	**1**

**Table 5:** Comparison of results on 100-dimensional non-fixed dimensional functions.

F(x)	Metric	GA	PSO	GWO	HHO	ACO	WOA	CMA-ES	SOGWO	EGWO	TACPSO	SCSO	MSCSO
F1	Mean	2.2796 × 10^05^	4.1309 × 10^03^	1.9649 × 10^−12^	8.1783 × 10^−94^	1.1445 × 10^05^	7.9144 × 10^−73^	1.7426 × 10^01^	3.0552 × 10^−12^	8.5587 × 10^−16^	6.2226 × 10^03^	8.4594 × 10^−104^	**0.0000 × 10^00^**
	Std	2.1903 × 10^04^	1.5709 × 10^03^	1.6092 × 10^−12^	4.4712 × 10^−93^	1.4191 × 10^04^	4.1414 × 10^−72^	3.4458 × 10^00^	2.3656 × 10^−12^	1.3989 × 10^−15^	2.2899 × 10^03^	4.4412 × 10^−103^	**0.0000 × 10^00^**
	P	1.2118 × 10^−12^	1.2118 × 10^−12^	1.2118 × 10^−12^	1.2118E−12	1.2118 × 10^−12^	1.2118 × 10^−12^	1.2118 × 10^−12^	1.2118 × 10^−12^	1.2118 × 10^−12^	1.2118 × 10^−12^	1.2118 × 10^−12^	——
	Wr	(+)	(+)	(+)	(+)	(+)	(+)	(+)	(+)	(+)	(+)	(+)	——
F2	Mean	2.6881 × 10^02^	7.8502 × 10^01^	4.3109 × 10^−08^	6.2289 × 10^−50^	2.8672 × 10^23^	1.4848 × 10^−49^	1.0948 × 10^01^	4.3769 × 10^−08^	1.0503 × 10^−10^	1.0912 × 10^02^	4.7898 × 10^−55^	**0.0000 × 10^00^**
	Std	1.6706 × 10^01^	1.7924 × 10^01^	1.6356 × 10^−08^	3.1766 × 10^−49^	1.2002 × 10^24^	6.6602 × 10^−49^	2.0627 × 10^00^	1.6609 × 10^−08^	8.7046 × 10^−11^	3.1014 × 10^01^	1.9093 × 10^−54^	**0.0000 × 10^00^**
	P	1.2118 × 10^−12^	1.2118 × 10^−12^	1.2118 × 10^−12^	1.2118 × 10^−12^	1.2118 × 10^−12^	1.2118 × 10^−12^	1.2118 × 10^−12^	1.2118 × 10^−12^	1.2118 × 10^−12^	1.2118 × 10^−12^	1.2118 × 10^−12^	——
	Wr	(+)	(+)	(+)	(+)	(+)	(+)	(+)	(+)	(+)	(+)	(+)	——
F3	Mean	6.0279 × 10^05^	1.2364 × 10^05^	8.1177 × 10^02^	9.7975 × 10^−65^	5.4162 × 10^05^	1.0311 × 10^06^	4.0662 × 10^05^	1.5336 × 10^03^	2.7234 × 10^04^	8.6711 × 10^04^	3.5996 × 10^−87^	**0.0000 × 10^00^**
	Std	1.6306 × 10^05^	4.8487 × 10^04^	1.2470 × 10^03^	5.2305 × 10^−64^	6.0352 × 10^04^	3.2664 × 10^05^	5.0995 × 10^04^	1.2227 × 10^03^	1.3463 × 10^04^	1.5929 × 10^04^	1.9623 × 10^−86^	**0.0000 × 10^00^**
	P	1.2118 × 10^−12^	1.2118 × 10^−12^	1.2118 × 10^−12^	1.2118 × 10^−12^	1.2118 × 10^−12^	1.2118 × 10^−12^	1.2118 × 10^−12^	1.2118 × 10^−12^	1.2118 × 10^−12^	1.2118 × 10^−12^	1.2118 × 10^−12^	——
	Wr	(+)	(+)	(+)	(+)	(+)	(+)	(+)	(+)	(+)	(+)	(+)	——
F4	Mean	9.3715 × 10^01^	2.2901 × 10^01^	1.2118 × 10^00^	4.1398 × 10^−48^	9.7205 × 10^01^	7.6390 × 10^01^	9.9136 × 10^01^	1.4083 × 10^00^	7.2801 × 10^01^	4.7416 × 10^01^	3.0384 × 10^−47^	**0.0000 × 10^00^**
	Std	2.6212 × 10^00^	5.0716 × 10^00^	1.8149 × 10^00^	1.5216 × 10^−47^	1.2321 × 10^00^	1.9532 × 10^01^	1.3058 × 10^00^	1.1685 × 10^00^	9.1623 × 10^00^	3.4754 × 10^00^	1.5472 × 10^−46^	**0.0000 × 10^00^**
	P	1.2118 × 10^−12^	1.2118 × 10^−12^	1.2118 × 10^−12^	1.2118 × 10^−12^	1.2118 × 10^−12^	1.2118 × 10^−12^	1.2118 × 10^−12^	1.2118 × 10^−12^	1.2118 × 10^−12^	1.2118 × 10^−12^	1.2118 × 10^−12^	——
	Wr	(+)	(+)	(+)	(+)	(+)	(+)	(+)	(+)	(+)	(+)	(+)	——
F5	Mean	8.8575 × 10^08^	2.9507 × 10^05^	9.7907 × 10^01^	**6.1032 × 10^−02^**	1.1344 × 10^09^	9.8113 × 10^01^	9.2200 × 10^02^	9.7793 × 10^01^	9.8304 × 10^01^	3.2176 × 10^06^	9.8589 × 10^01^	8.1960 × 10^−01^
	Std	1.2162 × 10^08^	1.7422 × 10^05^	7.6201 × 10^−01^	**9.6318 × 10^−02^**	2.1787 × 10^08^	2.7765 × 10^−01^	6.9787 × 10^02^	8.1377 × 10^−01^	5.2734 × 10^−01^	2.0883 × 10^06^	1.9354 × 10^−01^	1.4538 × 10^00^
	P	3.0199 × 10^−11^	3.0199 × 10^−11^	3.0199 × 10^−11^	1.0188 × 10^−05^	3.0199 × 10^−11^	3.0199 × 10^−11^	3.0199 × 10^−11^	3.0199 × 10^−11^	3.0199 × 10^−11^	3.0199 × 10^−11^	3.0199 × 10^−11^	——
	Wr	(+)	(+)	(+)	(−)	(+)	(+)	(+)	(+)	(+)	(+)	(+)	——
F6	Mean	2.2850 × 10^05^	4.0293 × 10^03^	9.9389 × 10^00^	**2.4948 × 10^−04^**	1.1967 × 10^05^	4.3402 × 10^00^	1.7082 × 10^01^	1.0292 × 10^01^	1.5039 × 10^01^	6.3488 × 10^03^	1.4374 × 10^01^	8.8979 × 10^−04^
	Std	2.1925 × 10^04^	1.4985 × 10^03^	9.6829 × 10^−01^	**2.8808 × 10^−04^**	1.4465 × 10^04^	1.3761 × 10^00^	3.0428 × 10^00^	9.8669 × 10^−01^	1.0101 × 10^00^	2.0877 × 10^03^	1.3427 × 10^00^	9.0667 × 10^−04^
	P	3.0199 × 10^−11^	3.0199 × 10^−11^	3.0199 × 10^−11^	3.3679 × 10^−04^	3.0199 × 10^−11^	3.0199 × 10^−11^	3.0199 × 10^−11^	3.0199 × 10^−11^	3.0199 × 10^−11^	3.0199 × 10^−11^	3.0199 × 10^−11^	——
	Wr	(+)	(+)	(+)	(−)	(+)	(+)	(+)	(+)	(+)	(+)	(+)	——
F7	Mean	1.2880 × 10^03^	1.8519 × 10^01^	7.3043 × 10^−03^	**1.4652 × 10^−04^**	8.3847 × 10^02^	4.8867 × 10^−03^	1.6478 × 10^−01^	5.9650 × 10^−03^	3.1838 × 10^−02^	1.4838 × 10^01^	2.7908 × 10^−04^	3.8534 × 10^−04^
	Std	2.7904 × 10^02^	3.4375 × 10^01^	3.3281 × 10^−03^	**1.2302 × 10^−04^**	2.7992 × 10^02^	5.4639 × 10^−03^	2.7897 × 10^−02^	2.2195 × 10^−03^	1.7459 × 10^−02^	1.3548 × 10^01^	5.8690 × 10^−04^	6.0105 × 10^−04^
	P	3.0199 × 10^−11^	3.0199 × 10^−11^	3.0199 × 10^−11^	2.9047 × 10^−01^	3.0199 × 10^−11^	2.3897 × 10^−08^	3.0199 × 10^−11^	3.3384 × 10^−11^	3.0199 × 10^−11^	3.0199 × 10^−11^	4.6427 × 10^−01^	——
	Wr	(+)	(+)	(+)	(=)	(+)	(+)	(+)	(+)	(+)	(+)	(=)	——
F8	Mean	4.6556 × 10^06^	1.0718 × 10^04^	8.7212 × 10^−03^	**1.3345 × 10^−04^**	3.1155 × 10^06^	4.0181 × 10^−03^	1.4693 × 10^00^	9.0207 × 10^−03^	3.5840 × 10^−02^	1.5888 × 10^04^	1.9658 × 10^−04^	3.2212 × 10^−04^
	Std	1.0644 × 10^06^	3.0968 × 10^04^	3.4001 × 10^−03^	**1.0575 × 10^−04^**	5.1347 × 10^05^	4.7751 × 10^−03^	4.9195 × 10^−01^	3.4850 × 10^−03^	2.0738 × 10^−02^	1.1767 × 10^04^	1.8249 × 10^−04^	7.1252 × 10^−04^
	P	3.0199 × 10^−11^	3.0199 × 10^−11^	3.0199 × 10^−11^	8.4180 × 10^−01^	3.0199 × 10^−11^	8.3520 × 10^−08^	3.0199 × 10^−11^	3.0199 × 10^−11^	3.0199 × 10^−11^	3.0199 × 10^−11^	5.1060 × 10^−01^	——
	Wr	(+)	(+)	(+)	(=)	(+)	(+)	(+)	(+)	(+)	(+)	(=)	——
F9	Mean	3.2977 × 10^−08^	1.5079 × 10^−09^	6.4755 × 10^−16^	**1.9287 × 10^−22^**	1.9287 × 10^−22^	**1.9287 × 10^−22^**	7.7732 × 10^−19^	1.1438 × 10^−15^	9.5797 × 10^−10^	1.1098 × 10^−21^	2.5733 × 10^−13^	**1.9287 × 10^−22^**
	Std	6.1594 × 10^−08^	3.0777 × 10^−09^	7.9144 × 10^−16^	**0.0000 × 10^00^**	1.0729 × 10^−36^	**0.0000 × 10^00^**	9.9456 × 10^−19^	2.5022 × 10^−15^	2.4510 × 10^−09^	2.0133 × 10^−21^	4.8276 × 10^−13^	**0.0000 × 10^00^**
	P	1.2118 × 10^−12^	1.1064 × 10^−12^	1.2118 × 10^−12^	NaN	1.2864 × 10^−08^	Na	1.2118 × 10^−12^	1.2118 × 10^−12^	1.2118 × 10^−12^	3.0208 × 10^−07^	1.2118 × 10^−12^	——
	Wr	(+)	(+)	(+)	(=)	(+)	(=)	(+)	(+)	(+)	(+)	(+)	——
F10	Mean	5.1483 × 10^82^	3.5975 × 10^51^	2.6735 × 10^−53^	3.4519 × 10^−121^	1.1690 × 10^73^	3.5429 × 10^−82^	9.3166 × 10^48^	5.8934 × 10^−51^	2.6623 × 10^27^	4.0074 × 10^44^	4.6375 × 10^−211^	**0.0000 × 10^00^**
	Std	1.8609 × 10^83^	1.8265 × 10^52^	1.4630 × 10^−52^	1.7459 × 10^−120^	5.1268 × 10^73^	1.9405 × 10^−81^	3.9737 × 10^49^	2.8320 × 10^−50^	1.4581 × 10^28^	1.8306 × 10^45^	0.0000 × 10^00^	**0.0000 × 10^00^**
	P	1.2118 × 10^−12^	1.2118 × 10^−12^	1.2118 × 10^−12^	1.2118 × 10^−12^	1.2118 × 10^−12^	1.2118 × 10^−12^	1.2118 × 10^−12^	1.2118 × 10^−12^	1.2118 × 10^−12^	1.2118 × 10^−12^	1.2118 × 10^−12^	——
	Wr	(+)	(+)	(+)	(+)	(+)	(+)	(+)	(+)	(+)	(+)	(+)	——
F11	Mean	3.9619 × 10^05^	4.1432 × 10^03^	7.0203 × 10^−13^	1.8974 × 10^−95^	5.3103 × 10^05^	4.1676 × 10^−72^	3.1191 × 10^00^	9.9913 × 10^−13^	9.9624 × 10^−16^	2.3445 × 10^03^	2.0590 × 10^−104^	**0.0000 × 10^00^**
	Std	8.6142 × 10^04^	9.7870 × 10^03^	5.6979 × 10^−13^	7.8619 × 10^−95^	1.1470 × 10^05^	1.9547 × 10^−71^	5.6696 × 10^−01^	8.3325 × 10^−13^	2.1026 × 10^−15^	9.3347 × 10^02^	1.1273 × 10^−103^	**0.0000 × 10^00^**
	P	1.2118 × 10^−12^	1.2118 × 10^−12^	1.2118 × 10^−12^	1.2118 × 10^−12^	1.2118 × 10^−12^	1.2118 × 10^−12^	1.2118 × 10^−12^	1.2118 × 10^−12^	1.2118 × 10^−12^	1.2118 × 10^−12^	1.2118 × 10^−12^	——
	Wr	(+)	(+)	(+)	(+)	(+)	(+)	(+)	(+)	(+)	(+)	(+)	——
F12	Mean	1.2109 × 10^06^	1.0441 × 10^04^	6.8895 × 10^−01^	**2.5098 × 10^−01^**	7.4605 × 10^05^	6.6762 × 10^−01^	2.0708 × 10^01^	6.6674 × 10^−01^	6.6668 × 10^−01^	5.1931 × 10^03^	6.6667 × 10^−01^	2.9749 × 10^−01^
	Std	1.7705 × 10^05^	2.2701 × 10^04^	8.4553 × 10^−02^	1.6496 × 10^−03^	2.4831 × 10^05^	9.8690 × 10^−04^	5.6741 × 10^00^	2.5311 × 10^−05^	2.2236 × 10^−05^	3.3124 × 10^03^	**6.3080 × 10^−07^**	8.4863 × 10^−02^
	P	3.0199 × 10^−11^	3.0199 × 10^−11^	3.0199 × 10^−11^	7.6973 × 10^−04^	3.0199 × 10^−11^	3.0199 × 10^−11^	3.0199 × 10^−11^	3.0199 × 10^−11^	3.0199 × 10^−11^	3.0199 × 10^−11^	3.0199 × 10^−11^	——
	Wr	(+)	(+)	(+)	(−)	(+)	(+)	(+)	(+)	(+)	(+)	(+)	——
F13	Mean	6.9563 × 10^06^	3.2556 × 10^00^	**0.0000 × 10^00^**	3.7898 × 10^−215^	**0.0000 × 10^00^**	**0.0000 × 10^00^**	7.8789 × 10^00^	**0.0000 × 10^00^**	**0.0000 × 10^00^**	1.7522 × 10^−66^	**0.0000 × 10^00^**	**0.0000 × 10^00^**
	Std	2.4021 × 10^07^	9.7576 × 10^00^	**0.0000 × 10^00^**	**0.0000 × 10^00^**	**0.0000 × 10^00^**	**0.0000 × 10^00^**	1.6600 × 10^01^	**0.0000 × 10^00^**	**0.0000 × 10^00^**	9.0765 × 10^−66^	**0.0000 × 10^00^**	**0.0000 × 10^00^**
	P	1.2118 × 10^−12^	1.2118 × 10^−12^	NaN	5.8522 × 10^−09^	NaN	NaN	1.2118 × 10^−12^	NaN	NaN	1.2118 × 10^−12^	NaN	——
	Wr	(+)	(+)	(=)	(+)	(=)	(=)	(+)	(=)	(=)	(+)	(=)	——
F14	Mean	1.0643 × 10^07^	1.1869 × 10^03^	1.5923 × 10^−202^	1.7476 × 10^−99^	2.1583 × 10^−296^	2.6076 × 10^−102^	2.7539 × 10^02^	5.1224 × 10^−62^	1.4005 × 10^−267^	4.5004 × 10^−58^	5.1791 × 10^−244^	**0.0000 × 10^00^**
	Std	3.3461 × 10^07^	2.9411 × 10^03^	**0.0000 × 10^00^**	9.5623 × 10^−99^	**0.0000 × 10^00^**	1.0957 × 10^−101^	3.0001 × 10^02^	2.8057 × 10^−61^	**0.0000 × 10^00^**	1.1347 × 10^−57^	**0.0000 × 10^00^**	**0.0000 × 10^00^**
	P	1.2118 × 10^−12^	1.2118 × 10^−12^	1.2118 × 10^−12^	1.2118 × 10^−12^	1.2118 × 10^−12^	1.2118 × 10^−12^	1.2118 × 10^−12^	1.2118 × 10^−12^	1.9457 × 10^−09^	1.2118 × 10^−12^	1.2118 × 10^−12^	——
	Wr	(+)	(+)	(+)	(+)	(+)	(+)	(+)	(+)	(+)	(+)	(+)	——
F15	Mean	−4.0513 × 10^03^	−1.4926 × 10^04^	−1.6092 × 10^04^	**−4.1896 × 10^04^**	−1.5725 × 10^04^	−3.5063 × 10^04^	−1.3104 × 10^04^	−1.6515 × 10^04^	−1.7161 × 10^04^	−2.2572 × 10^04^	−1.9187 × 10^04^	−4.1892 × 10^04^
	Std	6.2481 × 10^02^	2.0901 × 10^03^	2.4194 × 10^03^	**3.3138 × 10^00^**	3.4541 × 10^03^	5.4556 × 10^03^	5.2254 × 10^02^	3.3694 × 10^03^	1.5809 × 10^03^	1.6226 × 10^03^	1.4736 × 10^03^	7.4988 × 10^00^
	P	3.0199 × 10^−11^	3.0199 × 10^−11^	3.0199 × 10^−11^	8.1200 × 10^−04^	3.0199 × 10^−11^	3.8202 × 10^−10^	2.9691 × 10^−11^	3.0199 × 10^−11^	3.0199 × 10^−11^	3.0199 × 10^−11^	3.0199 × 10^−11^	——
	Wr	(+)	(+)	(+)	(−)	(+)	(+)	(+)	(+)	(+)	(+)	(+)	——
F16	Mean	1.5305 × 10^03^	8.7163 × 10^02^	1.0704 × 10^01^	**0.0000 × 10^00^**	1.4030 × 10^03^	2.2737 × 10^−14^	9.1420 × 10^02^	9.2606 × 10^00^	8.3838 × 10^02^	4.5994 × 10^02^	**0.0000 × 10^00^**	**0.0000 × 10^00^**
	Std	7.4282 × 10^01^	9.8737 × 10^01^	6.0539 × 10^00^	**0.0000 × 10^00^**	4.1987 × 10^01^	6.9378 × 10^−14^	3.3052 × 10^01^	5.2711 × 10^00^	1.5836 × 10^02^	6.3770 × 10^01^	**0.0000 × 10^00^**	**0.0000 × 10^00^**
	P	1.2118 × 10^−12^	1.2118 × 10^−12^	1.2118 × 10^−12^	NaN	1.2118 × 10^−12^	8.1404 × 10^−02^	1.2118 × 10^−12^	1.2118 × 10^−12^	1.2118 × 10^−12^	1.2118 × 10^−12^	NaN	——
	Wr	(+)	(+)	(+)	(=)	(+)	(−)	(+)	(+)	(+)	(+)	(=)	——
F17	Mean	2.0813 × 10^01^	8.9150 × 10^00^	1.1883 × 10^−07^	**8.8818 × 10^−16^**	2.0778 × 10^01^	4.4409 × 10^−15^	1.8915 × 10^01^	1.3312 × 10^−07^	6.8008 × 10^−09^	1.2287 × 10^01^	**8.8818 × 10^−16^**	**8.8818 × 10^−16^**
	Std	1.1073 × 10^−01^	3.2976 × 10^00^	4.2129 × 10^−08^	**0.0000 × 10^00^**	3.3228 × 10^−02^	2.2853 × 10^−15^	4.3504 × 10^00^	4.9113 × 10^−08^	5.1643 × 10^−09^	9.6392 × 10^−01^	**0.0000 × 10^00^**	**0.0000 × 10^00^**
	P	1.2118 × 10^−12^	1.2118 × 10^−12^	1.2118 × 10^−12^	NaN	1.2118 × 10^−12^	9.8401 × 10^−10^	1.2118 × 10^−12^	1.2118 × 10^−12^	1.2118 × 10^−12^	1.2118 × 10^−12^	NaN	——
	Wr	(+)	(+)	(+)	(=)	(+)	(+)	(+)	(+)	(+)	(+)	(=)	——
F18	Mean	2.0005 × 10^03^	3.6336 × 10^01^	5.6536 × 10^−03^	**0.0000 × 10^00^**	1.0152 × 10^03^	**0.0000 × 10^00^**	1.1646 × 10^00^	9.6033 × 10^−03^	7.9317 × 10^−03^	5.8580 × 10^01^	**0.0000 × 10^00^**	**0.0000 × 10^00^**
	Std	2.3542 × 10^02^	1.5089 × 10^01^	1.2226 × 10^−02^	**0.0000 × 10^00^**	1.3432 × 10^02^	**0.0000 × 10^00^**	2.2550 × 10^−02^	1.3643 × 10^−02^	1.2348 × 10^−02^	1.9599 × 10^01^	**0.0000 × 10^00^**	**0.0000 × 10^00^**
	P	1.2118 × 10^−12^	1.2118 × 10^−12^	1.2118 × 10^−12^	NaN	1.2118 × 10^−12^	NaN	1.2118 × 10^−12^	1.2118 × 10^−12^	1.9154 × 10^−09^	1.2118 × 10^−12^	NaN	——
	Wr	(+)	(+)	(+)	(=)	(+)	(=)	(+)	(+)	(+)	(+)	(=)	——
F19	Mean	1.7363 × 10^09^	1.0009 × 10^03^	3.0634 × 10^−01^	**3.7823 × 10^−06^**	3.1386 × 10^09^	4.9345 × 10^−02^	3.2312 × 10^−01^	2.8489 × 10^−01^	1.5560 × 10^01^	6.9676 × 10^04^	3.4836 × 10^−01^	1.3032 × 10^−05^
	Std	4.2025 × 10^08^	5.2710 × 10^03^	7.2027 × 10^−02^	**4.4886 × 10^−06^**	2.8856 × 10^08^	2.2000 × 10^−02^	1.2786 × 10^−01^	7.0244 × 10^−02^	1.0702 × 10^01^	9.3768 × 10^04^	9.5973 × 10^−02^	1.5304 × 10^−05^
	P	3.0199 × 10^−11^	3.0199 × 10^−11^	3.0199 × 10^−11^	3.1830 × 10^−03^	3.0199 × 10^−11^	3.0199 × 10^−11^	3.0199 × 10^−11^	3.0199 × 10^−11^	3.0199 × 10^−11^	3.0199 × 10^−11^	3.0199 × 10^−11^	——
	Wr	(+)	(+)	(+)	(−)	(+)	(+)	(+)	(+)	(+)	(+)	(+)	——
F20	Mean	3.5933 × 10^09^	5.6724 × 10^04^	6.9081 × 10^00^	**1.5929 × 10^−04^**	5.5965 × 10^09^	2.7952 × 10^00^	4.0591 × 10^00^	6.8193 × 10^00^	2.7049 × 10^01^	2.3673 × 10^06^	9.7397 × 10^00^	4.4916 × 10^−04^
	Std	5.9842 × 10^08^	8.4445 × 10^04^	4.2975 × 10^−01^	**1.5891 × 10^−04^**	6.1779 × 10^08^	8.5577 × 10^−01^	1.0288 × 10^00^	4.0145 × 10^−01^	4.2160 × 10^01^	2.7130 × 10^06^	9.3944 × 10^−02^	6.4329 × 10^−04^
	P	3.0199 × 10^−11^	3.0199 × 10^−11^	3.0199 × 10^−11^	1.2235 × 10^−01^	3.0199 × 10^−11^	3.0199 × 10^−11^	3.0199 × 10^−11^	3.0199 × 10^−11^	3.0199 × 10^−11^	3.0199 × 10^−11^	3.0199 × 10^−11^	——
	Wr	(+)	(+)	(+)	(=)	(+)	(+)	(+)	(+)	(+)	(+)	(+)	——
Wilcoxon’s rank sum test		20/0/0	20/0/0	19/1/0	8/7/5	19/1/0	16/3/1	20/0/0	19/1/0	19/1/0	20/0/0	14/6/0	——
Friedman value		1.1150 × 10^01^	9.4750 × 10^00^	5.4375 × 10^00^	2.4500 × 10^00^	9.7250 × 10^00^	5.0250 × 10^00^	8.2250 × 10^00^	5.7625 × 10^00^	6.2875 × 10^00^	8.8000 × 10^00^	3.4500 × 10^00^	**2.2125 × 10^00^**
Friedman rank		12	10	5	2	11	4	8	6	7	9	3	**1**

**Table 6:** Comparison of results on 500-dimensional non-fixed dimensional functions.

F(x)	Metric	GA	PSO	GWO	HHO	ACO	WOA	CMA-ES	SOGWO	EGWO	TACPSO	SCSO	MSCSO
F1	Mean	1.4977 × 10^06^	3.1397 × 10^04^	1.7613 × 10^−03^	8.8328 × 10^−95^	1.5648 × 10^06^	2.9543 × 10^−68^	2.2126 × 10^05^	2.2573 × 10^−03^	1.0849 × 10^−05^	2.9514 × 10^05^	5.6077 × 10^−97^	**0.0000 × 10^00^**
	Std	4.4331 × 10^04^	8.5563 × 10^03^	5.5599 × 10^−04^	3.3822 × 10^−94^	3.5002 × 10^04^	1.6151 × 10^−67^	1.6771 × 10^04^	8.5204 × 10^−04^	1.2365 × 10^−05^	1.9603 × 10^04^	2.7758 × 10^−96^	**0.0000 × 10^00^**
	P	1.2118 × 10^−12^	1.2118 × 10^−12^	1.2118 × 10^−12^	1.2118 × 10^−12^	1.2118 × 10^−12^	1.2118 × 10^−12^	1.2118 × 10^−12^	1.2118 × 10^−12^	1.2118 × 10^−12^	1.2118 × 10^−12^	1.2118 × 10^−12^	——
	Wr	(+)	(+)	(+)	(+)	(+)	(+)	(+)	(+)	(+)	(+)	(+)	——
F2	Mean	5.4992 × 10^222^	4.2782 × 10^02^	1.1083 × 10^−02^	3.5377 × 10^−47^	1.7181 × 10^268^	2.5354 × 10^−49^	1.0609 × 10^150^	4.0119 × 10^150^	1.3815 × 10^−04^	4.2159 × 10^23^	2.0238 × 10^−52^	**0.0000 × 10^00^**
	Std	Inf	7.8024 × 10^01^	1.7558 × 10^−03^	1.6076 × 10^−46^	Inf	1.0333 × 10^−48^	4.0119 × 10^150^	1.6260 × 10^−03^	7.1073 × 10^−05^	2.2984 × 10^24^	3.7943 × 10^−52^	**0.0000 × 10^00^**
	P	1.2118 × 10^−12^	1.2118 × 10^−12^	1.2118 × 10^−12^	1.2118 × 10^−12^	1.2118 × 10^−12^	1.2118 × 10^−12^	1.2118 × 10^−12^	1.2118 × 10^−12^	1.2118 × 10^−12^	1.2118 × 10^−12^	1.2118 × 10^−12^	——
	Wr	(+)	(+)	(+)	(+)	(+)	(+)	(+)	(+)	(+)	(+)	(+)	——
F3	Mean	1.9802 × 10^07^	3.0740 × 10^06^	3.2480 × 10^05^	9.0454 × 10^−46^	1.3429 × 10^07^	3.0265 × 10^07^	9.0761 × 10^06^	3.6470 × 10^05^	1.4732 × 10^06^	2.0009 × 10^06^	6.2146 × 10^−81^	**0.0000 × 10^00^**
	Std	8.0170 × 10^06^	1.8959 × 10^06^	8.2783 × 10^04^	4.9542 × 10^−45^	2.0759 × 10^06^	1.0334 × 10^07^	8.7328 × 10^05^	9.2667 × 10^04^	2.5059 × 10^05^	5.0885 × 10^05^	3.3915 × 10^−80^	**0.0000 × 10^00^**
	P	1.2118 × 10^−12^	1.2118 × 10^−12^	1.2118 × 10^−12^	1.2118 × 10^−12^	1.2118 × 10^−12^	1.2118 × 10^−12^	1.2118 × 10^−12^	1.2118 × 10^−12^	1.2118 × 10^−12^	1.2118 × 10^−12^	1.2118 × 10^−12^	——
	Wr	(+)	(+)	(+)	(+)	(+)	(+)	(+)	(+)	(+)	(+)	(+)	——
F4	Mean	9.9138 × 10^01^	3.5549 × 10^01^	6.4807 × 10^01^	1.2872 × 10^−48^	9.9396 × 10^01^	8.2266 × 10^01^	9.9812 × 10^01^	6.6963 × 10^01^	9.7072 × 10^01^	6.9371 × 10^01^	2.1131 × 10^−44^	**0.0000 × 10^00^**
	Std	2.6133 × 10^−01^	4.8545 × 10^00^	4.0676 × 10^00^	4.1619 × 10^−48^	2.8396 × 10^−01^	2.4726 × 10^01^	2.4606 × 10^−01^	5.0286 × 10^00^	6.4644 × 10^−01^	1.7810 × 10^00^	1.1365 × 10^−43^	**0.0000 × 10^00^**
	P	1.2118 × 10^−12^	1.2118 × 10^−12^	1.2118 × 10^−12^	1.2118 × 10^−12^	1.2118 × 10^−12^	1.2118 × 10^−12^	1.2118 × 10^−12^	1.2118 × 10^−12^	1.2118 × 10^−12^	1.2118 × 10^−12^	1.2118 × 10^−12^	——
	Wr	(+)	(+)	(+)	(+)	(+)	(+)	(+)	(+)	(+)	(+)	(+)	——
F5	Mean	6.8192 × 10^09^	1.0362 × 10^07^	4.9804 × 10^02^	**1.6816 × 10^−01^**	4.9891 × 10^02^	4.9631 × 10^02^	3.5042 × 10^08^	4.9807 × 10^02^	9.7400 × 10^04^	4.2102 × 10^08^	4.9845 × 10^02^	2.0871 × 10^01^
	Std	2.8680 × 10^08^	6.4980 × 10^06^	2.6747 × 10^−01^	**2.8584 × 10^−01^**	4.1249 × 10^−02^	3.8186 × 10^−01^	5.0317 × 10^07^	3.8521 × 10^−01^	3.0459 × 10^05^	4.9563 × 10^07^	8.9807 × 10^−02^	5.0497 × 10^01^
	P	3.0199 × 10^−11^	3.0199 × 10^−11^	3.0199 × 10^−11^	4.6856 × 10^−08^	3.0199 × 10^−11^	3.0199 × 10^−11^	3.0199 × 10^−11^	3.0199 × 10^−11^	3.0199 × 10^−11^	3.0199 × 10^−11^	3.0199 × 10^−11^	——
	Wr	(+)	(+)	(+)	(−)	(+)	(+)	(+)	(+)	(+)	(+)	(+)	——
F6	Mean	1.4913 × 10^06^	3.5389 × 10^04^	9.1566 × 10^01^	**3.7310 × 10^−03^**	1.5608 × 10^06^	3.2604 × 10^01^	2.2310 × 10^05^	9.1592 × 10^01^	1.0576 × 10^02^	3.0073 × 10^05^	1.0674 × 10^02^	7.0995 × 10^−03^
	Std	4.2928 × 10^04^	1.0628 × 10^04^	2.2706 × 10^00^	**4.2920 × 10^−03^**	4.1429 × 10^04^	9.6870 × 10^00^	1.2747 × 10^04^	1.8252 × 10^00^	1.4150 × 10^00^	1.4538 × 10^04^	2.7812 × 10^00^	1.1931 × 10^−02^
	P	3.0199 × 10^−11^	3.0199 × 10^−11^	3.0199 × 10^−11^	1.3732E−01	3.0199 × 10^−11^	3.0199 × 10^−11^	3.0199 × 10^−11^	3.0199 × 10^−11^	3.0199 × 10^−11^	3.0199 × 10^−11^	3.0199 × 10^−11^	——
	Wr	(+)	(+)	(+)	(=)	(+)	(+)	(+)	(+)	(+)	(+)	(+)	——
F7	Mean	5.6255 × 10^04^	2.5865 × 10^03^	5.0710 × 10^−02^	2.3710 × 10^−04^	6.0085 × 10^04^	3.9607 × 10^−03^	4.7686 × 10^03^	4.5808 × 10^−02^	2.1429 × 10^00^	6.0645 × 10^03^	**1.1311 × 10^−04^**	4.8096 × 10^−04^
	Std	2.6073 × 10^03^	1.7603 × 10^03^	1.2792 × 10^−02^	4.0484 × 10^−04^	2.5759 × 10^03^	4.7070 × 10^−03^	4.9426 × 10^02^	9.6847 × 10^−03^	2.0928 × 10^00^	1.1985 × 10^03^	**9.3870 × 10^−05^**	9.7120 × 10^−04^
	P	3.0199 × 10^−11^	3.0199 × 10^−11^	3.0199 × 10^−11^	7.5059 × 10^−01^	3.0199 × 10^−11^	5.0723 × 10^−10^	3.0199 × 10^−11^	3.0199 × 10^−11^	3.0199 × 10^−11^	3.0199 × 10^−11^	3.6322 × 10^−01^	——
	Wr	(+)	(+)	(+)	(=)	(+)	(+)	(+)	(+)	(+)	(+)	(=)	——
F8	Mean	2.0767 × 10^08^	1.1618 × 10^06^	1.1913 × 10^−01^	**1.8727 × 10^−04^**	2.2269 × 10^08^	3.6961 × 10^−03^	1.8301 × 10^07^	1.2687 × 10^−01^	4.4363 × 10^03^	1.2149 × 10^07^	2.7154 × 10^−04^	4.1224 × 10^−04^
	Std	1.0727 × 10^07^	1.1187 × 10^06^	2.7234 × 10^−02^	**1.5671 × 10^−04^**	7.6520 × 10^06^	5.6367 × 10^−03^	2.4005 × 10^06^	2.3678 × 10^−02^	2.2041 × 10^04^	1.6307 × 10^06^	4.9701 × 10^−04^	6.7304 × 10^−04^
	P	3.0199 × 10^−11^	3.0199 × 10^−11^	3.0199 × 10^−11^	2.3399 × 10^−01^	3.0199 × 10^−11^	1.0277 × 10^−06^	1.6980 × 10^−08^	3.0199 × 10^−11^	3.0199 × 10^−11^	3.0199 × 10^−11^	2.5805 × 10^−01^	——
	Wr	(+)	(+)	(+)	(=)	(+)	(+)	(+)	(+)	(+)	(+)	(=)	——
F9	Mean	1.7925 × 10^−10^	1.3682 × 10^−18^	2.0314 × 10^−38^	**2.6692 × 10^−109^**	2.2041 × 10^−108^	**2.6692 × 10^−109^**	7.8137 × 10^−65^	3.8006 × 10^−38^	6.4597 × 10^−24^	8.7207 × 10^−101^	3.7782 × 10^−30^	**2.6692 × 10^−109^**
	Std	9.1302 × 10^−10^	5.7709 × 10^−18^	5.5846 × 10^−38^	**9.6162 × 10^−125^**	3.4167 × 10^−108^	**9.6162 × 10^−125^**	2.0501 × 10^−64^	1.5688 × 10^−37^	3.1494 × 10^−23^	4.7480 × 10^−100^	2.0638 × 10^−29^	**9.6162 × 10^−125^**
	P	1.2118 × 10^−12^	1.2118 × 10^−12^	1.2000 × 10^−12^	NaN	1.2118 × 10^−12^	NaN	1.2118 × 10^−12^	1.2118 × 10^−12^	1.2118 × 10^−12^	1.1651 × 10^−12^	1.2118 × 10^−12^	——
	Wr	(+)	(+)	(+)	(=)	(+)	(=)	(+)	(+)	(+)	(+)	(+)	——
F10	Mean	Inf	Inf	3.6934 × 10^10^	4.0490 × 10^−119^	Inf	1.2129 × 10^−101^	Inf	2.5286 × 10^−04^	3.7059 × 10^207^	3.3333 × 10^232^	1.3386 × 10^−210^	**0.0000 × 10^00^**
	Std	NaN	NaN	2.0229 × 10^11^	2.2168 × 10^−118^	NaN	6.6401 × 10^−101^	NaN	1.3850 × 10^−03^	Inf	Inf	**0.0000 × 10^00^**	**0.0000 × 10^00^**
	P	1.6853 × 10^−14^	1.6853 × 10^−14^	1.2118 × 10^−12^	1.2118 × 10^−12^	1.6853 × 10^−14^	1.2118 × 10^−12^	1.6853 × 10^−14^	1.2118 × 10^−12^	1.2118 × 10^−12^	1.2118 × 10^−12^	1.2118 × 10^−12^	——
	Wr	(+)	(+)	(+)	(+)	(+)	(+)	(+)	(+)	(+)	(+)	(+)	——
F11	Mean	1.5676 × 10^07^	3.6029 × 10^05^	7.0041 × 10^−03^	2.0151 × 10^−94^	1.6670 × 10^07^	4.9140 × 10^−70^	8.8429 × 10^05^	6.5687 × 10^−03^	2.2807 × 10^00^	8.7724 × 10^00^	1.2842 × 10^−97^	**0.0000 × 10^00^**
	Std	6.3020 × 10^05^	3.2012 × 10^05^	2.3146 × 10^−03^	2.0151 × 10^−94^	6.6729 × 10^05^	1.8243 × 10^−69^	1.0381 × 10^05^	2.4959 × 10^−03^	8.7724 × 10^00^	1.3034 × 10^05^	4.7136 × 10^−97^	**0.0000 × 10^00^**
	P	1.2118 × 10^−12^	1.2118 × 10^−12^	1.2118 × 10^−12^	1.2118 × 10^−12^	1.2118 × 10^−12^	1.2118 × 10^−12^	1.2118 × 10^−12^	1.2118 × 10^−12^	1.2118 × 10^−12^	1.2118 × 10^−12^	1.2118 × 10^−12^	——
	Wr	(+)	(+)	(+)	(+)	(+)	(+)	(+)	(+)	(+)	(+)	(+)	——
F12	Mean	5.2208 × 10^07^	1.0206 × 10^06^	8.1360 × 10^−01^	**2.5151 × 10^−01^**	5.6751 × 10^07^	7.0253 × 10^−01^	4.8663 × 10^06^	8.8203 × 10^−01^	1.7687 × 10^02^	3.4207 × 10^06^	6.6667 × 10^−01^	7.0904 × 10^−01^
	Std	2.0914 × 10^06^	7.9598 × 10^05^	1.5772 × 10^−01^	3.7954 × 10^−03^	1.7916 × 10^06^	7.5690 × 10^−02^	5.1206 × 10^05^	1.5918 × 10^−01^	5.5992 × 10^02^	3.4454 × 10^05^	**7.6552 × 10^−07^**	7.0904 × 10^−01^
	P	3.0199 × 10^−11^	3.0199 × 10^−11^	8.7710 × 10^−02^	5.5727 × 10^−10^	3.0199 × 10^−11^	1.9073 × 10^−01^	3.0199 × 10^−11^	6.5486 × 10^−04^	1.0702 × 10^−09^	3.0199 × 10^−11^	7.7272 × 10^−02^	——
	Wr	(+)	(+)	(=)	(−)	(+)	(=)	(+)	(+)	(+)	(+)	(=)	——
F13	Mean	5.5514 × 10^06^	1.7456 × 10^00^	**0.0000 × 10^00^**	3.8344 × 10^−204^	**0.0000 × 10^00^**	**0.0000 × 10^00^**	1.1121 × 10^01^	**0.0000 × 10^00^**	**0.0000 × 10^00^**	4.6247 × 10^−67^	**0.0000 × 10^00^**	**0.0000 × 10^00^**
	Std	7.7247 × 10^06^	4.0383 × 10^00^	**0.0000 × 10^00^**	**0.0000 × 10^00^**	**0.0000 × 10^00^**	**0.0000 × 10^00^**	2.0778 × 10^01^	**0.0000 × 10^00^**	**0.0000 × 10^00^**	1.8707 × 10^−66^	**0.0000 × 10^00^**	**0.0000 × 10^00^**
	P	1.2118 × 10^−12^	1.2118 × 10^−12^	NaN	1.9346 × 10^−10^	NaN	NaN	1.2118 × 10^−12^	NaN	NaN	1.2118 × 10^−12^	NaN	——
	Wr	(+)	(+)	(=)	(+)	(=)	(=)	(+)	(=)	(=)	(+)	(=)	——
F14	Mean	8.2125 × 10^06^	4.5534 × 10^02^	1.9021 × 10^−206^	2.5887 × 10^−100^	6.4825 × 10^−294^	4.6056 × 10^−102^	3.6800 × 10^02^	2.7047 × 10^−47^	2.8033 × 10^−256^	1.6283 × 10^−57^	4.7963 × 10^−237^	**0.0000 × 10^00^**
	Std	1.3622 × 10^07^	1.6572 × 10^03^	**0.0000 × 10^00^**	1.4145 × 10^−99^	**0.0000 × 10^00^**	2.3305 × 10^−101^	2.6847 × 10^02^	1.1742 × 10^−46^	**0.0000 × 10^00^**	5.5162 × 10^−57^	**0.0000 × 10^00^**	**0.0000 × 10^00^**
	P	1.2118 × 10^−12^	1.2118 × 10^−12^	1.2118 × 10^−12^	1.2118 × 10^−12^	1.2118 × 10^−12^	1.2118 × 10^−12^	1.2118 × 10^−12^	1.2118 × 10^−12^	1.9346 × 10^−10^	1.2118 × 10^−12^	1.2118 × 10^−12^	——
	Wr	(+)	(+)	(+)	(+)	(+)	(+)	(+)	(+)	(+)	(+)	(+)	——
F15	Mean	−9.4627 × 10^03^	−3.5233 × 10^04^	−5.4762 × 10^04^	**−2.0948 × 10^05^**	−3.0797 × 10^04^	−1.8122 × 10^05^	−2.2446 × 10^04^	−5.2633 × 10^04^	−4.7961 × 10^04^	−6.3985 × 10^04^	−6.1348 × 10^04^	−2.0944 × 10^05^
	Std	1.7324 × 10^03^	5.0990 × 10^03^	1.2302 × 10^04^	**1.8715 × 10^01^**	6.6733 × 10^03^	2.6623 × 10^04^	2.2163 × 10^03^	1.3708 × 10^04^	4.3140 × 10^03^	4.5266 × 10^03^	3.6742 × 10^03^	8.2445 × 10^01^
	P	3.0199 × 10^−11^	3.0199 × 10^−11^	3.0199 × 10^−11^	2.1506 × 10^−02^	3.0199 × 10^−11^	9.9186 × 10^−11^	3.0199 × 10^−11^	3.0199 × 10^−11^	3.0199 × 10^−11^	3.0199 × 10^−11^	3.0199 × 10^−11^	——
	Wr	(+)	(+)	(+)	(−)	(+)	(+)	(+)	(+)	(+)	(+)	(+)	——
F16	Mean	8.7350 × 10^03^	4.2617 × 10^03^	6.9260 × 10^01^	**0.0000 × 10^00^**	8.8896 × 10^03^	**0.0000 × 10^00^**	6.9922 × 10^03^	9.7784 × 10^01^	5.2329 × 10^03^	4.4655 × 10^03^	**0.0000 × 10^00^**	**0.0000 × 10^00^**
	Std	1.0567 × 10^02^	4.7422 × 10^02^	1.8520 × 10^01^	**0.0000 × 10^00^**	1.3020 × 10^02^	**0.0000 × 10^00^**	9.7784 × 10^01^	2.6807 × 10^01^	1.0524 × 10^03^	1.5555 × 10^02^	**0.0000 × 10^00^**	**0.0000 × 10^00^**
	P	1.2118 × 10^−12^	1.2118 × 10^−12^	1.2118 × 10^−12^	NaN	1.2118 × 10^−12^	NaN	1.2118 × 10^−12^	1.2118 × 10^−12^	1.2118 × 10^−12^	1.2118 × 10^−12^	NaN	——
	Wr	(+)	(+)	(+)	(=)	(+)	(=)	(+)	(+)	(+)	(+)	(=)	——
F17	Mean	2.1104 × 10^01^	1.1179 × 10^01^	1.7992 × 10^−03^	**8.8818 × 10^−16^**	2.1013 × 10^01^	5.2699 × 10^−15^	2.0988 × 10^01^	1.9941 × 10^−03^	1.4581 × 10^−04^	1.8243 × 10^01^	**8.8818 × 10^−16^**	**8.8818 × 10^−16^**
	Std	2.7540 × 10^−02^	3.2017 × 10^00^	3.0725 × 10^−04^	**0.0000 × 10^00^**	1.1582 × 10^−02^	2.4120 × 10^−15^	1.4466 × 10^−02^	3.8350 × 10^−04^	1.0052 × 10^−04^	1.7898 × 10^−01^	**0.0000 × 10^00^**	**0.0000 × 10^00^**
	P	1.2118 × 10^−12^	1.2078 × 10^−12^	1.2118 × 10^−12^	NaN	1.2118 × 10^−12^	1.1003 × 10^−10^	1.2118 × 10^−12^	1.2118 × 10^−12^	1.2118 × 10^−12^	1.2118 × 10^−12^	NaN	——
	Wr	(+)	(+)	(+)	(=)	(+)	(+)	(+)	(+)	(+)	(+)	(=)	——
F18	Mean	1.3531 × 10^04^	3.0519 × 10^02^	1.1862 × 10^−02^	**0.0000 × 10^00^**	1.4049 × 10^04^	**0.0000 × 10^00^**	1.9821 × 10^03^	4.1634 × 10^−02^	1.2083 × 10^−02^	2.6489 × 10^03^	**0.0000 × 10^00^**	**0.0000 × 10^00^**
	Std	3.4343 × 10^02^	8.1281 × 10^01^	3.1222 × 10^−02^	**0.0000 × 10^00^**	4.0180 × 10^02^	**0.0000 × 10^00^**	1.3456 × 10^02^	5.8013 × 10^−02^	2.6952 × 10^−02^	1.2709 × 10^02^	**0.0000 × 10^00^**	**0.0000 × 10^00^**
	P	1.2118 × 10^−12^	1.2118 × 10^−12^	1.2118 × 10^−12^	NaN	1.2118 × 10^−12^	NaN	1.2118 × 10^−12^	1.2118 × 10^−12^	1.2118 × 10^−12^	1.2118 × 10^−12^	NaN	——
	Wr	(+)	(+)	(+)	(=)	(+)	(=)	(+)	(+)	(+)	(+)	(=)	——
F19	Mean	1.6871 × 10^10^	2.3391 × 10^05^	2.7477 × 10^05^	**3.2586 × 10^−06^**	1.8114 × 10^10^	9.9567 × 10^−02^	5.1739 × 10^08^	7.9653 × 10^−01^	4.3263 × 10^07^	3.5742 × 10^08^	7.8758 × 10^−01^	7.6443 × 10^−06^
	Std	8.2188 × 10^08^	2.7477 × 10^05^	6.3557 × 10^−02^	**4.7260 × 10^−06^**	8.1213 × 10^08^	4.9571 × 10^−02^	1.1508 × 10^08^	7.9933 × 10^−02^	4.7311 × 10^07^	6.4163 × 10^07^	7.2447 × 10^−02^	8.5768 × 10^−06^
	P	3.0199 × 10^−11^	3.0199 × 10^−11^	3.0199 × 10^−11^	9.0688 × 10^−03^	3.0199 × 10^−11^	3.0199 × 10^−11^	3.0199 × 10^−11^	3.0199 × 10^−11^	3.0199 × 10^−11^	3.0199 × 10^−11^	3.0199 × 10^−11^	——
	Wr	(+)	(+)	(+)	(−)	(+)	(+)	(+)	(+)	(+)	(+)	(+)	——
F20	Mean	3.0778 × 10^10^	4.4311 × 10^06^	5.0740 × 10^01^	**4.7357 × 10^−04^**	3.2917 × 10^10^	1.8085 × 10^01^	1.1541 × 10^09^	5.1221 × 10^01^	1.2245 × 10^07^	1.7574 × 10^07^	4.9816 × 10^01^	2.0716 × 10^−03^
	Std	1.4788 × 10^09^	3.3146 × 10^06^	1.6573 × 10^00^	**6.1694 × 10^−04^**	1.5376 × 10^09^	7.5770 × 10^00^	1.8369 × 10^08^	1.8298 × 10^00^	1.7574 × 10^07^	1.7874 × 10^08^	7.2371 × 10^−02^	3.0945 × 10^−03^
	P	3.0199 × 10^−11^	3.0199 × 10^−11^	3.0199 × 10^−11^	8.1200 × 10^−04^	3.0199 × 10^−11^	3.0199 × 10^−11^	3.0199 × 10^−11^	3.0199 × 10^−11^	3.0199 × 10^−11^	3.0199 × 10^−11^	3.0199 × 10^−11^	——
	Wr	(+)	(+)	(+)	(−)	(+)	(+)	(+)	(+)	(+)	(+)	(+)	——
Wilcoxon’s rank sum test		20/0/0	20/0/0	18/2/0	8/7/5	19/1/0	15/5/0	20/0/0	19/1/0	19/1/0	20/0/0	13/7/0	——
Friedman value		1.0888 × 10^01^	8.7250 × 10^00^	5.4375 × 10^00^	2.5625 × 10^00^	9.4750 × 10^00^	4.6625 × 10^00^	9.1750 × 10^00^	6.3125 × 10^00^	6.7000 × 10^00^	8.5875 × 10^00^	3.2250 × 10^00^	**2.2500 × 10^00^**
Friedman rank		12	9	5	2	11	4	10	6	7	8	3	**1**

**Table 7:** Comparison of results on fixed-dimensional functions.

F(x)	Metric	GA	PSO	GWO	HHO	ACO	WOA	CMA-ES	SOGWO	EGWO	TACPSO	SCSO	MSCSO
F21	Mean	9.9996 × 10^−01^	**9.9800 × 10^−01^**	4.2992 × 10^00^	1.6235 × 10^00^	4.1334 × 10^00^	3.6486 × 10^00^	7.4200 × 10^00^	3.2311 × 10^00^	8.8986 × 10^00^	**9.9800 × 10^−01^**	4.5586 × 10^00^	**9.9800 × 10^−01^**
	Std	1.0163 × 10^−02^	1.6020 × 10^−10^	3.8844 × 10^00^	1.5213 × 10^00^	4.0030 × 10^00^	3.5083 × 10^00^	4.1400 × 10^00^	2.6989 × 10^00^	5.0473 × 10^00^	**1.4867 × 10^−16^**	4.0421 × 10^00^	2.5409 × 10^−12^
	P	3.6897 × 10^−11^	2.6099 × 10^−10^	9.9186 × 10^−11^	3.3384 × 10^−11^	1.8398 × 10^−01^	4.0772 × 10^−11^	3.0199 × 10^−11^	3.0199 × 10^−11^	3.4548 × 10^−10^	7.7632 × 10^−12^	7.6950 × 10^−08^	——
	Wr	(+)	(+)	(+)	(+)	(=)	(+)	(+)	(+)	(+)	(−)	(+)	——
F22	Mean	1.6642 × 10^−02^	1.0024 × 10^−02^	3.2080 × 10^−03^	**3.7552 × 10^−04^**	4.0808 × 10^−03^	7.9626 × 10^−04^	5.3984 × 10^−03^	7.7841 × 10^−03^	8.5570 × 10^−03^	1.1908 × 10^−03^	4.7696 × 10^−04^	4.9924 × 10^−04^
	Std	1.9314 × 10^−02^	9.8328 × 10^−03^	6.8552 × 10^−03^	**1.7199 × 10^−04^**	7.4081 × 10^−03^	5.5383 × 10^−04^	3.8866 × 10^−03^	9.6429 × 10^−03^	1.3336 × 10^−02^	3.6376 × 10^−03^	3.5300 × 10^−04^	3.3424 × 10^−04^
	P	4.9752 × 10^−11^	1.3289 × 10^−10^	5.1877 × 10^−02^	7.9585 × 10^−01^	1.6057 × 10^−06^	3.8481 × 10^−03^	3.0199 × 10^−11^	3.9881 × 10^−04^	2.0095 × 10^−01^	1.5013 × 10^−02^	7.4827 × 10^−02^	——
	Wr	(+)	(+)	(=)	(=)	(+)	(+)	(+)	(+)	(=)	(−)	(=)	——
F23	Mean	−9.7347 × 10^−01^	−1.0316 × 10^00^	−1.0316 × 10^00^	−1.0316 × 10^00^	−1.0316 × 10^00^	−1.0316 × 10^00^	−1.0316 × 10^00^	−1.0316 × 10^00^	−1.0316 × 10^00^	**−1.0316 × 10^00^**	−1.0316 × 10^00^	−1.0316 × 10^00^
	Std	7.7637 × 10^−02^	3.1472 × 10^−05^	4.3488 × 10^−08^	2.5625 × 10^−09^	6.7752 × 10^−16^	3.0772 × 10^−09^	6.7752 × 10^−16^	2.6709 × 10^−08^	4.0944 × 10^−09^	**6.0459 × 10^−16^**	7.3591 × 10^−10^	5.3275 × 10^−08^
	P	3.0199 × 10^−11^	3.6897 × 10^−11^	4.4440 × 10^−07^	9.0292 × 10^−04^	1.2118 × 10^−12^	2.8913 × 10^−03^	1.2118 × 10^−12^	1.8682 × 10^−05^	4.1279 × 10^−03^	1.2455 × 10^−11^	5.7460 × 10^−02^	——
	Wr	(+)	(+)	(+)	(−)	(−)	(−)	(−)	(+)	(+)	(−)	(=)	——
F24	Mean	3.9902 × 10^−01^	3.9789 × 10^−01^	3.9789 × 10^−01^	3.9789 × 10^−01^	**3.9789 × 10^−01^**	3.9790 × 10^−01^	**3.9789 × 10^−01^**	3.9789 × 10^−01^	3.9789 × 10^−01^	**3.9789 × 10^−01^**	3.9789 × 10^−01^	3.9789 × 10^−01^
	Std	2.3114 × 10^−03^	4.4680 × 10^−06^	1.1344 × 10^−06^	1.8372 × 10^−06^	**0.0000 × 10^00^**	1.5164 × 10^−05^	**0.0000 × 10^00^**	8.6789 × 10^−07^	1.6174 × 10^−07^	**0.0000 × 10^00^**	2.2747 × 10^−08^	5.6349 × 10^−08^
	P	3.6897 × 10^−11^	1.3289 × 10^−10^	2.4386 × 10^−09^	4.1127 × 10^−07^	1.2118 × 10^−12^	9.9186 × 10^−11^	1.2118 × 10^−12^	4.6159 × 10^−10^	6.7362 × 10^−06^	1.2118 × 10^−12^	9.4696 × 10^−01^	——
	Wr	(+)	(+)	(+)	(+)	(−)	(+)	(−)	(+)	(+)	(−)	(=)	——
F25	Mean	5.6019 × 10^00^	3.0003 × 10^00^	3.0000 × 10^00^	3.0000 × 10^00^	6.6000 × 10^00^	3.0001 × 10^00^	5.4112 × 10^00^	5.7000 × 10^00^	1.1368 × 10^01^	**3.0000 × 10^00^**	3.0000 × 10^00^	3.0004 × 10^00^
	Std	7.1539 × 10^00^	4.8541 × 10^−04^	3.6279 × 10^−05^	2.4443 × 10^−06^	1.5426 × 10^01^	2.2400 × 10^−04^	1.3207 × 10^01^	1.4789 × 10^01^	2.5562 × 10^01^	**2.2281 × 10^−15^**	1.0066 × 10^−05^	1.6404 × 10^−03^
	P	6.2828 × 10^−06^	1.7666 × 10^−03^	2.0621 × 10^−01^	8.1014 × 10^−10^	3.5048 × 10^−09^	7.3940 × 10^−01^	1.4516 × 10^−10^	1.9579 × 10^−01^	1.0315 × 10^−02^	2.7391 × 10^−11^	3.6709 × 10^−03^	——
	Wr	(+)	(+)	(=)	(−)	(−)	(=)	(−)	(=)	(−)	(−)	(−)	——
F26	Mean	−3.2300 × 10^00^	−3.8601 × 10^00^	−3.8621 × 10^00^	−3.8593 × 10^00^	−3.8628 × 10^00^	−3.8544 × 10^00^	−3.8628 × 10^00^	−3.8607 × 10^00^	−3.8618 × 10^00^	**−3.8628 × 10^00^**	−3.8618 × 10^00^	−3.8618 × 10^00^
	Std	4.4069 × 10^−01^	3.7334 × 10^−03^	1.8444 × 10^−03^	4.3845 × 10^−03^	2.7101 × 10^−15^	1.5473 × 10^−02^	2.7101 × 10^−15^	3.1028 × 10^−03^	2.5789 × 10^−03^	**2.5684 × 10^−15^**	2.4887 × 10^−03^	2.6529 × 10^−03^
	P	3.0199 × 10^−11^	5.2978 × 10^−01^	9.2344 × 10^−01^	4.7138 × 10^−04^	1.2118 × 10^−12^	2.6695 × 10^−09^	1.2118 × 10^−12^	2.0621 × 10^−01^	2.4157 × 10^−02^	1.1364 × 10^−11^	1.0763 × 10^−02^	——
	Wr	(+)	(=)	(=)	(+)	(−)	(+)	(−)	(=)	(−)	(−)	(−)	——
F27	Mean	−1.4744 × 10^00^	−3.0142 × 10^00^	−3.2249 × 10^00^	−3.0907 × 10^00^	**−3.2784 × 10^00^**	−3.2359 × 10^00^	−3.1633 × 10^00^	−3.2538 × 10^00^	−3.2658 × 10^00^	−3.2604 × 10^00^	−3.1612 × 10^00^	−3.2607 × 10^00^
	Std	5.5810 × 10^−01^	−3.0142 × 10^00^	1.0807 × 10^−01^	9.3938 × 10^−02^	**5.8273 × 10^−02^**	9.1771 × 10^−02^	1.8828 × 10^−01^	8.2493 × 10^−02^	8.0172 × 10^−02^	6.3773 × 10^−02^	2.1967 × 10^−01^	7.4356 × 10^−02^
	P	3.0199 × 10^−11^	5.1857 × 10^−07^	8.3026 × 10^−01^	2.0152 × 10^−08^	7.6093 × 10^−05^	3.8481 × 10^−03^	3.7449 × 10^−01^	1.6687 × 10^−01^	9.0490 × 10^−02^	6.1841 × 10^−03^	4.6427 × 10^−01^	——
	Wr	(+)	(+)	(=)	(+)	(−)	(+)	(=)	(=)	(=)	(−)	(=)	——
F28	Mean	−9.1447 × 10^−01^	−8.5278 × 10^00^	−9.0605 × 10^00^	−5.5277 × 10^00^	−5.4185 × 10^00^	−7.9164 × 10^00^	−7.3444 × 10^00^	−9.0563 × 10^00^	−6.0541 × 10^00^	−6.6356 × 10^00^	−5.2589 × 10^00^	**−1.0153 × 10^01^**
	Std	6.9185 × 10^−01^	2.5091 × 10^00^	2.2568 × 10^00^	1.4563 × 10^00^	3.6642 × 10^00^	2.7743 × 10^00^	3.2173 × 10^00^	2.2649 × 10^00^	3.3166 × 10^00^	3.2628 × 10^00^	1.5331 × 10^00^	**1.5412 × 10^−04^**
	P	3.0199 × 10^−11^	5.9615 × 10^−09^	4.0772 × 10^−11^	3.0199 × 10^−11^	7.3554 × 10^−02^	3.0199 × 10^−11^	6.6056 × 10^−01^	6.0658 × 10^−11^	6.6955 × 10^−11^	3.7805 × 10^−01^	5.5727 × 10^−10^	——
	Wr	(+)	(+)	(+)	(+)	(=)	(+)	(=)	(+)	(+)	(=)	(+)	——
F29	Mean	−9.9176 × 10^−01^	−9.0792 × 10^00^	−9.8709 × 10^00^	−5.2550 × 10^00^	−7.0297 × 10^00^	−8.4873 × 10^00^	−9.1620 × 10^00^	−1.0401 × 10^01^	−7.9763 × 10^00^	−7.9478 × 10^00^	−6.9606 × 10^00^	**−1.0403 × 10^01^**
	Std	4.9834 × 10^−01^	2.4464 × 10^00^	1.6171 × 10^00^	9.3970 × 10^−01^	3.6826 × 10^00^	3.0282 × 10^00^	2.8266 × 10^00^	8.0404 × 10^−04^	3.5316 × 10^00^	3.3520 × 10^00^	2.6969 × 10^00^	**3.6603 × 10^−05^**
	P	3.0199 × 10^−11^	1.3854 × 10^−09^	3.0199 × 10^−11^	3.0199 × 10^−11^	6.6168 × 10^−01^	4.0772 × 10^−11^	7.8548 × 10^−06^	3.0199 × 10^−11^	4.5043 × 10^−11^	7.4445 × 10^−02^	4.9980 × 10^−09^	——
	Wr	(+)	(+)	(+)	(+)	(=)	(+)	(−)	(+)	(+)	(=)	(+)	——
F30	Mean	−1.4199 × 10^00^	−1.0508 × 10^01^	−1.0354 × 10^01^	−5.0779 × 10^00^	−8.3101 × 10^00^	−7.4034 × 10^00^	−9.4552 × 10^00^	−9.9938 × 10^00^	−6.7009 × 10^00^	−8.1710 × 10^00^	−6.0465 × 10^00^	**−1.0536 × 10^01^**
	Std	7.8499 × 10^−01^	3.8371 × 10^−02^	9.8702 × 10^−01^	2.4010 × 10^−01^	3.5013 × 10^00^	3.2711 × 10^00^	2.8037 × 10^00^	2.0583 × 10^00^	3.9600 × 10^00^	3.2254 × 10^00^	2.3410 × 10^00^	**3.5501 × 10^−05^**
	P	3.0199 × 10^−11^	1.0808 × 10^−10^	3.0199 × 10^−11^	3.0199 × 10^−11^	6.8412 × 10^−03^	3.0199 × 10^−11^	3.7124 × 10^−07^	3.0199 × 10^−11^	3.3384 × 10^−11^	7.6240 × 10^−02^	1.3111 × 10^−08^	——
	Wr	(+)	(+)	(+)	(+)	(−)	(+)	(−)	(+)	(+)	(=)	(+)	——
Wilcoxon’s rank sum test		10/0/0	9/1/0	6/4/0	7/1/2	1/3/6	8/1/1	2/2/6	7/3/0	6/2/2	0/3/7	4/4/2	——
Friedman value		9.4500 × 10^00^	6.2250 × 10^00^	5.5000 × 10^00^	6.0000 × 10^00^	6.8750 × 10^00^	7.2500 × 10^00^	6.4750 × 10^00^	6.2250 × 10^00^	8.5250 × 10^00^	4.5500 × 10^00^	6.5500 × 10^00^	**4.3750 × 10^00^**
Friedman rank		12	5	3	4	9	10	7	5	11	2	8	**1**

**Table 8:** Classification accuracy results on feature selection datasets.

Dataset	Metric	BACO	BBOA	BHHO	BWOA	BGWO	BGA	BPSO	BMSCSO
N1	Mean	**9.8333 × 10^−01^**	**9.8333 × 10^−01^**	**9.8333 × 10^−01^**	9.8000 × 10^−01^	**9.8333 × 10^−01^**	**9.8333 × 10^−01^**	**9.8333 × 10^−01^**	**9.8333 × 10^−01^**
	Std	1.7568E−02	1.7568 × 10^−02^	1.7568 × 10^−02^	1.7213 × 10^−02^	1.7568 × 10^−02^	1.7568 × 10^−02^	1.7568 × 10^−02^	1.7568 × 10^−02^
N2	Mean	9.4000 × 10^−01^	9.1857 × 10^−01^	9.0571 × 10^−01^	9.3714 × 10^−01^	**9.5286 × 10^−01^**	9.3571 × 10^−01^	9.3143 × 10^−01^	9.4571 × 10^−01^
	Std	3.0713 × 10^−02^	3.4372 × 10^−02^	4.4772 × 10^−02^	3.8803 × 10^−02^	3.6916 × 10^−02^	3.8832 × 10^−02^	4.4057 × 10^−02^	3.2156 × 10^−02^
N3	Mean	9.7000 × 10^−01^	9.2000 × 10^−01^	9.4000 × 10^−01^	9.4000 × 10^−01^	9.6000 × 10^−01^	9.6500 × 10^−01^	9.7000 × 10^−01^	**9.8500 × 10^−01^**
	Std	3.4960 × 10^−02^	2.5820 × 10^−02^	3.9441 × 10^−02^	3.9441 × 10^−02^	3.9441 × 10^−02^	4.1164 × 10^−02^	3.4960 × 10^−02^	2.4152 × 10^−02^
N4	Mean	**9.6571 × 10^−01^**	9.2571 × 10^−01^	9.2857 × 10^−01^	9.0857 × 10^−01^	9.5714 × 10^−01^	9.5429 × 10^−01^	9.5714 × 10^−01^	**9.6571 × 10^−01^**
	Std	2.6255 × 10^−02^	2.4094 × 10^−02^	3.8686 × 10^−02^	8.2808 × 10^−02^	4.3121 × 10^−02^	3.3537 × 10^−02^	3.3672 × 10^−02^	2.6255 × 10^−02^
N5	Mean	**7.5238 × 10^−01^**	7.1429 × 10^−01^	7.0714 × 10^−01^	7.2381 × 10^−01^	7.3810 × 10^−01^	7.3571 × 10^−01^	7.4048 × 10^−01^	**7.5238 × 10^−01^**
	Std	6.3690 × 10^−02^	7.0093 × 10^−02^	5.6176 × 10^−02^	5.9603 × 10^−02^	6.2492 × 10^−02^	7.2261 × 10^−02^	6.1935 × 10^−02^	6.3690 × 10^−02^
N6	Mean	9.1684 × 10^−01^	9.0526 × 10^−01^	9.1263 × 10^−01^	9.1158 × 10^−01^	**9.6421 × 10^−01^**	**9.6421 × 10^−01^**	9.4842 × 10^−01^	9.2737 × 10^−01^
	Std	2.5520 × 10^−02^	3.1773 × 10^−02^	2.9377 × 10^−02^	3.5158 × 10^−02^	2.5879 × 10^−02^	1.8699 × 10^−02^	2.8699 × 10^−02^	2.5520 × 10^−02^
N7	Mean	8.7963 × 10^−01^	8.3704 × 10^−01^	8.5000 × 10^−01^	8.4630 × 10^−01^	8.4815 × 10^−01^	8.7037 × 10^−01^	8.6667 × 10^−01^	**8.8519 × 10^−01^**
	Std	3.7293 × 10^−02^	6.1605 × 10^−02^	2.3827 × 10^−02^	4.2811 × 10^−02^	6.2830 × 10^−02^	3.3810 × 10^−02^	2.2764 × 10^−02^	3.1232 × 10^−02^
N8	Mean	9.1034 × 10^−01^	8.6207 × 10^−01^	8.5862 × 10^−01^	8.8621 × 10^−01^	9.1379 × 10^−01^	9.0345 × 10^−01^	8.8276 × 10^−01^	**9.1724 × 10^−01^**
	Std	2.9078 × 10^−02^	2.8155 × 10^−02^	4.4368 × 10^−02^	4.3161 × 10^−02^	2.4383 × 10^−02^	5.0887 × 10^−02^	3.3314 × 10^−02^	3.3314 × 10^−02^
N9	Mean	9.4615 × 10^−01^	9.3077 × 10^−01^	9.3077 × 10^−01^	9.3846 × 10^−01^	9.3333 × 10^−01^	9.3333 × 10^−01^	9.3590 × 10^−01^	**9.5385 × 10^−01^**
	Std	1.4555 × 10^−02^	1.2386 × 10^−02^	2.9731 × 10^−02^	2.1622 × 10^−02^	1.7928 × 10^−02^	2.4772 × 10^−02^	2.7695 × 10^−02^	2.3562 × 10^−02^
N10	Mean	9.7950 × 10^−01^	7.4950 × 10^−01^	7.5000 × 10^−01^	8.0400 × 10^−01^	9.0400 × 10^−01^	9.7200 × 10^−01^	9.1500 × 10^−01^	**9.8850 × 10^−01^**
	Std	3.3702 × 10^−02^	6.4354 × 10^−02^	7.4498 × 10^−02^	7.5085 × 10^−02^	1.2449 × 10^−01^	8.8544 × 10^−02^	1.1350 × 10^−01^	1.1316 × 10^−02^
N11	Mean	**9.8705 × 10^−01^**	9.7842 × 10^−01^	9.8129 × 10^−01^	9.8201 × 10^−01^	9.8129 × 10^−01^	9.8345 × 10^−01^	9.8561 × 10^−01^	**9.8705 × 10^−01^**
	Std	8.1676 × 10^−03^	1.3566 × 10^−02^	9.1001 × 10^−03^	1.0858 × 10^−02^	9.1001 × 10^−03^	1.0751 × 10^−02^	8.3072 × 10^−03^	8.1676 × 10^−03^
N12	Mean	9.6283 × 10^−01^	9.5398 × 10^−01^	9.5044 × 10^−01^	9.5487 × 10^−01^	9.6018 × 10^−01^	9.5487 × 10^−01^	9.5221 × 10^−01^	**9.6372 × 10^−01^**
	Std	1.3060 × 10^−02^	1.4925 × 10^−02^	1.7301 × 10^−02^	1.4116 × 10^−02^	1.5185 × 10^−02^	2.0202 × 10^−02^	1.4571 × 10^−02^	1.3486 × 10^−02^
N13	Mean	9.2737 × 10^−01^	9.0526 × 10^−01^	9.2526 × 10^−01^	9.2316 × 10^−01^	9.6105 × 10^−01^	**9.6947 × 10^−01^**	9.5579 × 10^−01^	9.5053 × 10^−01^
	Std	2.9125 × 10^−02^	2.8070 × 10^−02^	2.2440 × 10^−02^	3.1403 × 10^−02^	2.1658 × 10^−02^	2.4536 × 10^−02^	2.7982 × 10^−02^	2.8527 × 10^−02^
N14	Mean	8.5714 × 10^−01^	8.0000 × 10^−01^	8.5714 × 10^−01^	8.5714 × 10^−01^	**9.2857 × 10^−01^**	8.7857 × 10^−01^	8.5000 × 10^−01^	8.7857 × 10^−01^
	Std	8.9087 × 10^−02^	1.1567 × 10^−01^	8.9087 × 10^−02^	1.1168 × 10^−01^	5.8321 × 10^−02^	9.5535 × 10^−02^	1.1394 × 10^−01^	9.5535 × 10^−02^
N15	Mean	8.4230 × 10^−01^	8.0540 × 10^−01^	8.3090 × 10^−01^	8.3710 × 10^−01^	8.3900 × 10^−01^	8.4000 × 10^−01^	8.3980 × 10^−01^	**8.4270 × 10^−01^**
	Std	7.4095 × 10^−03^	1.1108 × 10^−02^	1.3076 × 10^−02^	8.5434 × 10^−03^	8.7560 × 10^−03^	7.2265 × 10^−03^	8.4564 × 10^−03^	7.1032 × 10^−03^
N16	Mean	9.0488 × 10^−01^	8.7805 × 10^−01^	8.8049 × 10^−01^	8.8780 × 10^−01^	**9.5854 × 10^−01^**	9.4390 × 10^−01^	9.3902 × 10^−01^	9.1951 × 10^−01^
	Std	2.4254 × 10^−02^	3.4493 × 10^−02^	3.1383 × 10^−02^	3.2924 × 10^−02^	2.3139 × 10^−02^	2.0080 × 10^−02^	3.4969 × 10^−02^	2.8281 × 10^−02^
N17	Mean	9.6782 × 10^−01^	9.5402 × 10^−01^	9.4943 × 10^−01^	9.5977 × 10^−01^	9.7356 × 10^−01^	9.6667 × 10^−01^	9.6322 × 10^−01^	9.6897 × 10^−01^
	Std	1.1871 × 10^−02^	2.0274 × 10^−02^	2.8774 × 10^−02^	2.1842 × 10^−02^	1.3328 × 10^−02^	2.0597 × 10^−02^	2.0845 × 10^−02^	1.3328 × 10^−02^
N18	Mean	7.6450 × 10^−01^	7.4200 × 10^−01^	7.4900 × 10^−01^	7.5650 × 10^−01^	**7.7550 × 10^−01^**	7.7200 × 10^−01^	7.7050 × 10^−01^	7.7350 × 10^−01^
	Std	1.6406 × 10^−02^	1.6700 × 10^−02^	2.2211 × 10^−02^	1.7329 × 10^−02^	1.7393 × 10^−02^	2.6055 × 10^−02^	1.6907 × 10^−02^	1.7167 × 10^−02^
N19	Mean	**7.6536 × 10^−01^**	7.4183 × 10^−01^	7.4379 × 10^−01^	7.5556 × 10^−01^	7.5948 × 10^−01^	7.6013 × 10^−01^	7.5425 × 10^−01^	**7.6536 × 10^−01^**
	Std	2.2314 × 10^−02^	1.9303 × 10^−02^	2.1960 × 10^−02^	2.0714 × 10^−02^	1.8435 × 10^−02^	2.1579 × 10^−02^	1.8792 × 10^−02^	2.2314 × 10^−02^
N20	Mean	9.7418 × 10^−01^	9.0407 × 10^−01^	9.6103 × 10^−01^	9.6651 × 10^−01^	9.6964 × 10^−01^	**9.8482 × 10^−01^**	9.6901 × 10^−01^	9.7653 × 10^−01^
	Std	7.2751 × 10^−03^	4.8269 × 10^−02^	1.0497 × 10^−02^	8.5779 × 10^−03^	1.1193 × 10^−02^	7.7391 × 10^−03^	1.3954 × 10^−02^	4.5476 × 10^−03^
N21	Mean	7.1667 × 10^−01^	6.9420 × 10^−01^	6.9203 × 10^−01^	6.9710 × 10^−01^	**7.2246 × 10^−01^**	7.1087 × 10^−01^	7.1304 × 10^−01^	7.1884 × 10^−01^
	Std	1.2528 × 10^−02^	1.5579 × 10^−02^	2.1127 × 10^−02^	1.2221 × 10^−02^	1.2340 × 10^−02^	2.4739 × 10^−02^	1.7148 × 10^−02^	1.7684 × 10^−02^
N22	Mean	**7.7869 × 10^−01^**	7.6557 × 10^−01^	7.6885 × 10^−01^	7.7541 × 10^−01^	7.7377 × 10^−01^	7.7213 × 10^−01^	**7.7869 × 10^−01^**	**7.7869 × 10^−01^**
	Std	3.2097 × 10^−02^	3.1910 × 10^−02^	3.1343 × 10^−02^	2.8967 × 10^−02^	3.4387 × 10^−02^	2.9376 × 10^−02^	3.2097 × 10^−02^	3.2097 × 10^−02^
N23	Mean	8.6866 × 10^−01^	8.1791 × 10^−01^	8.5672 × 10^−01^	8.6716 × 10^−01^	8.6716 × 10^−01^	8.6866 × 10^−01^	8.6269 × 10^−01^	**8.7015 × 10^−01^**
	Std	3.0507 × 10^−02^	5.9619 × 10^−02^	4.1146 × 10^−02^	3.1817 × 10^−02^	3.4790 × 10^−02^	2.9684 × 10^−02^	3.9676 × 10^−02^	2.9892 × 10^−02^
N24	Mean	**8.4036 × 10^−01^**	8.3735 × 10^−01^	8.3313 × 10^−01^	8.3675 × 10^−01^	8.3795 × 10^−01^	8.3976 × 10^−01^	**8.4036 × 10^−01^**	**8.4036 × 10^−01^**
	Std	3.2781 × 10^−02^	3.3117 × 10^−02^	2.9655 × 10^−02^	3.5746 × 10^−02^	3.4131 × 10^−02^	3.3745 × 10^−02^	3.2781 × 10^−02^	3.2781 × 10^−02^

**Table 9:** Friedman’s test results of classification accuracy on feature selection datasets.

Dataset	BACO	BBOA	BHHO	BWOA	BGWO	BGA	BPSO	BMSCSO
N1	**4**	**4**	**4**	8	**4**	**4**	**4**	**4**
N2	3	7	8	4	**1**	5	6	2
N3	2.5	8	6.5	6.5	5	4	2.5	**1**
N4	**1.5**	7	6	8	3.5	5	3.5	**1.5**
N5	**1.5**	7	8	6	4	5	3	**1.5**
N6	5	8	6	7	**1.5**	**1.5**	3	4
N7	2	8	5	7	6	3	4	**1**
N8	3	7	8	5	2	4	6	**1**
N9	2	7.5	7.5	3	5.5	5.5	4	**1**
N10	2	8	7	6	5	3	4	**1**
N11	**1.5**	8	6.5	5	6.5	4	3	**1.5**
N12	2	6	8	4.5	3	4.5	7	**1**
N13	5	8	6	7	2	**1**	3	4
N14	5	8	5	5	**1**	2.5	7	2.5
N15	2	8	7	6	5	3	4	**1**
N16	5	8	7	6	**1**	2	3	4
N17	3	7	8	6	**1**	4	5	2
N18	5	8	7	6	**1**	3	4	2
N19	**1.5**	8	7	5	4	3	6	**1.5**
N20	3	8	7	6	4	**1**	5	2
N21	3	7	8	6	**1**	5	4	2
N22	**2**	8	7	4	5	6	**2**	**2**
N23	2.5	8	7	4.5	4.5	2.5	6	**1**
N24	**2**	6	8	7	5	4	**2**	**2**
Sum of ranks	69	177.5	164.5	138.5	81.5	85.5	101	46.5
Sum of ranks squared	4761	31,506.25	27,060.25	19,182.25	6642.25	7310.25	10201	2162.25
Average of ranks	2.8750	7.3958	6.8542	5.7708	3.3958	3.5625	4.2083	1.9375

**Table 10:** Fitness results on feature selection datasets.

Dataset	Metric	BACO	BBOA	BHHO	BWOA	BGWO	BGA	BPSO	BMSCSO
N1	Mean	**2.0750 × 10^−02^**	2.1500 × 10^−02^	2.1500 × 10^−02^	2.4800 × 10^−02^	**2.0750 × 10^−02^**	2.1250 × 10^−02^	2.1000 × 10^−02^	**2.0750 × 10^−02^**
	Std	1.7210 × 10^−02^	1.7472 × 10^−02^	1.7989 × 10^−02^	1.7689 × 10^−02^	1.7210 × 10^−02^	1.7750 × 10^−02^	1.7504 × 10^−02^	1.7210 × 10^−02^
N2	Mean	6.0459 × 10^−02^	8.2908 × 10^−02^	9.5755 × 10^−02^	6.3846 × 10^−02^	**4.8260 × 10^−02^**	6.6496 × 10^−02^	7.0856 × 10^−02^	5.5890 × 10^−02^
	Std	3.0147 × 10^−02^	3.3392 × 10^−02^	4.4206 × 10^−02^	3.7801 × 10^−02^	3.6420 × 10^−02^	3.8245 × 10^−02^	4.3603 × 10^−02^	3.1943 × 10^−02^
N3	Mean	3.4075 × 10^−02^	8.3263 × 10^−02^	6.4650 × 10^−02^	6.3150 × 10^−02^	4.2850 × 10^−02^	3.7963 × 10^−02^	3.3638 × 10^−02^	**1.9288 × 10^−02^**
	Std	3.3048 × 10^−02^	2.5051 × 10^−02^	3.8308 × 10^−02^	3.7395 × 10^−02^	3.8556 × 10^−02^	3.9903 × 10^−02^	3.4338 × 10^−02^	2.3161 × 10^−02^
N4	Mean	**3.7276 × 10^−02^**	7.6710 × 10^−02^	7.3714 × 10^−02^	9.3681 × 10^−02^	4.5262 × 10^−02^	4.8007 × 10^−02^	4.5929 × 10^−02^	3.7776 × 10^−02^
	Std	2.5626 × 10^−02^	2.3663 × 10^−02^	3.8177 × 10^−02^	8.1330 × 10^−02^	4.3066 × 10^−02^	3.3071 × 10^−02^	3.3196 × 10^−02^	2.6817 × 10^−02^
N5	Mean	**2.4925 × 10^−01^**	2.8741 × 10^−01^	2.9482 × 10^−01^	2.7787 × 10^−01^	2.6295 × 10^−01^	2.6553 × 10^−01^	2.6137 × 10^−01^	2.4937 × 10^−01^
	Std	6.2291 × 10^−02^	6.8497 × 10^−02^	5.5994 × 10^−02^	5.7787 × 10^−02^	6.1638 × 10^−02^	7.1237 × 10^−02^	6.0426 × 10^−02^	6.2452 × 10^−02^
N6	Mean	8.6609 × 10^−02^	9.7187 × 10^−02^	8.9784 × 10^−02^	9.2832 × 10^−02^	**3.7938 × 10^−02^**	3.8817 × 10^−02^	5.5394 × 10^−02^	7.6658 × 10^−02^
	Std	2.4783 × 10^−02^	3.1049 × 10^−02^	2.9625 × 10^−02^	3.4197 × 10^−02^	2.5889 × 10^−02^	1.8765 × 10^−02^	2.8520 × 10^−02^	2.5526 × 10^−02^
N7	Mean	1.2247 × 10^−01^	1.6433 × 10^−01^	1.5158 × 10^−01^	1.5571 × 10^−01^	1.5333 × 10^−01^	1.3210 × 10^−01^	1.3477 × 10^−01^	**1.1759 × 10^−01^**
	Std	3.5687 × 10^−02^	6.0503 × 10^−02^	2.3815 × 10^−02^	4.2621 × 10^−02^	6.2034 × 10^−02^	3.3254 × 10^−02^	2.2460 × 10^−02^	3.0119 × 10^−02^
N8	Mean	9.2481 × 10^−02^	1.4016 × 10^−01^	1.4358 × 10^−01^	1.1566 × 10^−01^	8.8345 × 10^−02^	9.9086 × 10^−02^	1.1946 × 10^−01^	**8.6153 × 10^−02^**
	Std	2.8815 × 10^−02^	2.7508 × 10^−02^	4.3007 × 10^−02^	4.2646 × 10^−02^	2.4154 × 10^−02^	5.0409 × 10^−02^	3.3471 × 10^−02^	3.2651 × 10^−02^
N9	Mean	5.4717 × 10^−02^	7.0993 × 10^−02^	7.1084 × 10^−02^	6.1969 × 10^−02^	6.7318 × 10^−02^	6.6955 × 10^−02^	6.5280 × 10^−02^	**4.7510 × 10^−02^**
	Std	1.4138 × 10^−02^	1.1693 × 10^−02^	2.9006 × 10^−02^	2.1395 × 10^−02^	1.8088 × 10^−02^	2.4493 × 10^−02^	2.7141 × 10^−02^	2.2621 × 10^−02^
N10	Mean	2.5526 × 10^−02^	2.5253 × 10^−01^	2.5250 × 10^−01^	2.0204 × 10^−01^	1.0004 × 10^−01^	3.2643 × 10^−02^	8.9381 × 10^−02^	**1.6616 × 10^−02^**
	Std	3.3737 × 10^−02^	6.2487 × 10^−02^	7.1911 × 10^−02^	7.5714 × 10^−02^	1.2376 × 10^−01^	8.8631 × 10^−02^	1.1273 × 10^−01^	1.1380 × 10^−02^
N11	Mean	1.8153 × 10^−02^	2.6478 × 10^−02^	2.4185 × 10^−02^	2.3139 × 10^−02^	2.2518 × 10^−02^	2.1715 × 10^−02^	1.9689 × 10^−02^	**1.7820 × 10^−02^**
	Std	8.1168 × 10^−03^	1.3261 × 10^−02^	9.2854 × 10^−03^	1.0615 × 10^−02^	9.2964 × 10^−03^	1.0488 × 10^−02^	7.9495 × 10^−03^	7.6715 × 10^−03^
N12	Mean	3.9930 × 10^−02^	4.9191 × 10^−02^	5.1629 × 10^−02^	4.6881 × 10^−02^	4.0658 × 10^−02^	4.5781 × 10^−02^	4.9776 × 10^−02^	**3.9220 × 10^−02^**
	Std	1.2495 × 10^−02^	1.4799 × 10^−02^	1.7656 × 10^−02^	1.4514 × 10^−02^	1.4897 × 10^−02^	1.9883 × 10^−02^	1.4003 × 10^−02^	1.3138 × 10^−02^
N13	Mean	7.6294 × 10^−02^	9.7263 × 10^−02^	7.8259 × 10^−02^	8.0505 × 10^−02^	4.1067 × 10^−02^	**3.3401 × 10^−02^**	4.8062 × 10^−02^	5.3943 × 10^−02^
	Std	2.8333 × 10^−02^	2.7740 × 10^−02^	2.1545 × 10^−02^	3.0766 × 10^−02^	2.1286 × 10^−02^	2.4403 × 10^−02^	2.7740 × 10^−02^	2.8149 × 10^−02^
N14	Mean	1.4285 × 10^−01^	1.9944 × 10^−01^	1.4249 × 10^−01^	1.4244 × 10^−01^	**7.2157 × 10^−02^**	1.2278 × 10^−01^	1.5229 × 10^−01^	1.2177 × 10^−01^
	Std	8.7565 × 10^−02^	1.1365 × 10^−01^	8.7845 × 10^−02^	1.1021 × 10^−01^	5.7467 × 10^−02^	9.4502 × 10^−02^	1.1271 × 10^−01^	9.4325 × 10^−02^
N15	Mean	1.6441 × 10^−01^	1.9884 × 10^−01^	1.7560 × 10^−01^	1.7013 × 10^−01^	1.6591 × 10^−01^	1.6540 × 10^−01^	1.6555 × 10^−01^	**1.6373 × 10^−01^**
	Std	6.9856 × 10^−03^	1.0952 × 10^−02^	1.2407 × 10^−02^	7.9506 × 10^−03^	8.4674 × 10^−03^	6.7921 × 10^−03^	7.9944 × 10^−03^	7.1104 × 10^−03^
N16	Mean	9.7071 × 10^−02^	1.2332 × 10^−01^	1.2197 × 10^−01^	1.1344 × 10^−01^	**4.3115 × 10^−02^**	5.7770 × 10^−02^	6.3966 × 10^−02^	8.3466 × 10^−02^
	Std	2.4255 × 10^−02^	3.3907 × 10^−02^	3.0706 × 10^−02^	3.3025 × 10^−02^	2.2877 × 10^−02^	2.0283 × 10^−02^	3.5103 × 10^−02^	2.7712 × 10^−02^
N17	Mean	3.4425 × 10^−02^	4.9455 × 10^−02^	5.3256 × 10^−02^	4.2453 × 10^−02^	**2.9297 × 10^−02^**	3.5812 × 10^−02^	4.0351 × 10^−02^	3.4474 × 10^−02^
	Std	1.2014 × 10^−02^	2.0127 × 10^−02^	2.9055 × 10^−02^	2.1762 × 10^−02^	1.2744 × 10^−02^	2.0038 × 10^−02^	2.0549 × 10^−02^	1.3335 × 10^−02^
N18	Mean	2.3719 × 10^−01^	2.5950 × 10^−01^	2.5278 × 10^−01^	2.4640 × 10^−01^	**2.2546 × 10^−01^**	2.2989 × 10^−01^	2.3150 × 10^−01^	2.2898 × 10^−01^
	Std	1.6083 × 10^−02^	1.5701 × 10^−02^	1.9984 × 10^−02^	1.7786 × 10^−02^	1.6847 × 10^−02^	2.5991 × 10^−02^	1.6296 × 10^−02^	1.7136 × 10^−02^
N19	Mean	**2.3654 × 10^−01^**	2.5971 × 10^−01^	2.5802 × 10^−01^	2.4700 × 10^−01^	2.4237 × 10^−01^	2.4210 × 10^−01^	2.4779 × 10^−01^	**2.3654 × 10^−01^**
	Std	2.1125 × 10^−02^	1.9679 × 10^−02^	2.0966 × 10^−02^	2.0618 × 10^−02^	1.7589 × 10^−02^	2.0959 × 10^−02^	1.8135 × 10^−02^	2.1032 × 10^−02^
N20	Mean	3.2424 × 10^−02^	9.9694 × 10^−02^	4.5327 × 10^−02^	4.2044 × 10^−02^	3.3667 × 10^−02^	**1.9556 × 10^−02^**	3.6148 × 10^−02^	2.9656 × 10^−02^
	Std	6.6407 × 10^−03^	4.7989 × 10^−02^	8.8712 × 10^−03^	8.5118 × 10^−03^	1.0476 × 10^−02^	7.3417 × 10^−03^	1.3914 × 10^−02^	4.0424 × 10^−03^
N21	Mean	2.8414 × 10^−01^	3.0474 × 10^−01^	3.0811 × 10^−01^	3.0294 × 10^−01^	**2.7790 × 10^−01^**	2.9045 × 10^−01^	2.8723 × 10^−01^	2.8135 × 10^−01^
	Std	1.1812 × 10^−02^	1.5496 × 10^−02^	2.1654 × 10^−02^	1.1677 × 10^−02^	1.2370 × 10^−02^	2.4283 × 10^−02^	1.7214 × 10^−02^	1.7065 × 10^−02^
N22	Mean	**2.2477 × 10^−01^**	2.3675 × 10^−01^	2.3384 × 10^−01^	2.2834 × 10^−01^	2.2930 × 10^−01^	2.3126 × 10^−01^	**2.2477 × 10^−01^**	**2.2477 × 10^−01^**
	Std	3.0760 × 10^−02^	3.0239 × 10^−02^	2.9796 × 10^−02^	2.7559 × 10^−02^	3.2866 × 10^−02^	2.8226 × 10^−02^	3.0760 × 10^−02^	3.0760 × 10^−02^
N23	Mean	**1.3512 × 10^−01^**	1.8670 × 10^−01^	1.4914 × 10^−01^	1.3894 × 10^−01^	1.3794 × 10^−01^	1.3717 × 10^−01^	1.4208 × 10^−01^	**1.3512 × 10^−01^**
	Std	3.0151 × 10^−02^	5.8644 × 10^−02^	3.9245 × 10^−02^	3.2795 × 10^−02^	3.4572 × 10^−02^	2.9483 × 10^−02^	3.8550 × 10^−02^	3.0151 × 10^−02^
N24	Mean	**1.6244 × 10^−01^**	1.6542 × 10^−01^	1.6980 × 10^−01^	1.6622 × 10^−01^	1.6443 × 10^−01^	1.6304 × 10^−01^	**1.6244 × 10^−01^**	**1.6244 × 10^−01^**
	Std	3.1679 × 10^−02^	3.1196 × 10^−02^	2.8157 × 10^−02^	3.4901 × 10^−02^	3.2767 × 10^−02^	3.2648 × 10^−02^	3.1679 × 10^−02^	3.1679 × 10^−02^

**Table 11:** Friedman test results of fitness results on feature selection datasets.

Dataset	BACO	BBOA	BHHO	BWOA	BGWO	BGA	BPSO	BMSCSO
N1	**2**	6.5	6.5	8	**2**	5	4	**2**
N2	3	7	8	4	**1**	5	6	2
N3	3	8	7	6	5	4	2	**1**
N4	**1**	7	6	8	3	5	4	**2**
N5	**1**	7	8	6	4	5	3	**2**
N6	5	8	6	7	**1**	**2**	3	4
N7	2	8	5	7	6	3	4	**1**
N8	3	7	8	5	2	4	6	**1**
N9	2	7	8	3	6	5	4	**1**
N10	2	8	7	6	5	3	4	**1**
N11	2	8	7	6	5	4	3	**1**
N12	2	6	8	5	3	4	7	**1**
N13	5	8	6	7	2	**1**	3	4
N14	6	8	5	4	**1**	3	7	2
N15	2	8	7	6	5	3	4	**1**
N16	5	8	7	6	**1**	2	3	4
N17	2	7	8	6	**1**	4	5	3
N18	5	8	7	6	**1**	3	4	2
N19	**1.5**	8	7	5	4	3	6	**1.5**
N20	3	8	7	6	4	**1**	5	2
N21	3	7	8	6	**1**	5	4	2
N22	**2**	8	7	4	5	6	**2**	**2**
N23	**1.5**	8	7	5	4	3	6	**1.5**
N24	**2**	6	8	7	5	4	**2**	**2**
Sum of ranks	66	179.5	168.5	139	77	87	101	46
Sum of ranks squared	4356	32,220.25	28,392.25	19,321	5929	7569	10,201	2116
Average of ranks	2.7500	7.4792	7.0208	5.7917	3.2083	3.6250	4.2083	1.9167

## Data Availability

The data used in this article is publicly available and has been explained in the main text.

## Appendix A

**Table A1:** The global optimization functions.

Name	Function	Dim	Range	$F_{min}$	Type
Sphere	$f_{1}(x)=\sum_{i=1}^{Dim}x_{i}^{2}$	30, 100, 500	[−100, 100]	0	Unimodal
Schwefel 2.22	$f_{2}(x)=\sum_{i=1}^{n}\left|x_{i}\right|+\prod_{i=1}^{n}\left|x_{i}\right|$	30, 100, 500	[−1.28, 1.28]	0	Unimodal
Schwefel 1.2	$f_{3}(x)=\sum_{i=1}^{n}{\left(\sum_{j-1}^{i}x_{j}\right)}^{2}$	30, 100, 500	[−100, 100]	0	Unimodal
Schwefel 2.21	$f_{4}(x)=\max_{i}\left\{\left|x_{i}\right|,1⩽i⩽n\right\}$	30, 100, 500	[−100, 100]	0	Unimodal
Rosenbroke	$f_{5}(x)=\sum_{i=1}^{n-1}\left[100{\left(x_{i+1}-x_{i}^{2}\right)}^{2}+{\left(x_{i}-1\right)}^{2}\right]$	30, 100, 500	[−30, 30]	0	Unimodal
Step	$\;f_{6}\left(x\right)=\overset{n}{\underset{i=1}{\sum }}{\left[x_{i}+0.5\right]}^{2}$	30, 100, 500	[−100, 100]	0	Unimodal
Quartic	$f_{7}(x)=\sum_{i=1}^{n}ix_{i}^{4}+random[0,1]$	30, 100, 500	[−1.28, 1.28]	0	Unimodal
Exponential	$f_{8}=exp(0.5\sum_{i=1}^{n}x_{i})$	30, 100, 500	[−10, 10]	0	Unimodal
Sum Power	$f_{9}=\sum_{i=1}^{n}{\left|x_{i}\right|}^{\left(i+1\right)}$	30, 100, 500	[−1, 1]	0	Unimodal
Sum Square	$f_{10}(x)=\sum_{i=1}^{n}n\cdot x_{i}^{2}$	30, 100, 500	[−10, 10]	0	Unimodal
Zakharov	$f_{11}=\sum_{i=1}^{n}x_{i}{}^{2}+{\left(\sum_{i=1}^{n}0.5ix_{i}\right)}^{2}+{\left(\sum_{i=1}^{n}0.5ix_{i}\right)}^{4}$	30, 100, 500	[−10, 10]	0	Unimodal
Trid	$f_{12}(x)={\left(x_{i}-1\right)}^{2}+\sum_{i=1}^{n}i\cdot {\left(2x_{i}{}^{2}-x_{i-1}\right)}^{2}$	30, 100, 500	[−10, 10]	0	Unimodal
Elliptic	$f_{13}=\sum_{i=1}^{n}{\left(10^{6}\right)}^{\left(i-1)/(n-1\right)}\cdot x_{i}{}^{2}$	30, 100, 500	[−100, 100]	0	Unimodal
Cigar	$f_{14}(x)=x_{1}{}^{2}+10^{6}\sum_{i=1}^{n}x_{i}{}^{2}$	30, 100, 500	[−100, 100]	0	Unimodal
Generalized Schwefel’s problem 2.26	$f_{15}(x)=\sum_{i=1}^{n}-x_{i}\sin(\sqrt{\left|x_{i}\right|})$	30, 100, 500	[−500, 500]	−418.9829 × *n*	Multimodal
Generalized Rastrigin’s Function	$f_{16}(x)=\sum_{i=1}^{n}\left[x_{i}^{2}-10\cos\left(2\pi x_{i}\right)+10\right]$	30, 100, 500	[−5.12, 5.12]	0	Multimodal
Ackley’s Function	$\begin{array}{l} f_{17}(x)=-20\exp(-0.2\sqrt{\frac{1}{n}\sum_{i=1}^{n}x_{i}^{2}})-\\ \exp\left(\frac{1}{n}\sum_{i=1}^{n}\cos\left(2\pi x_{i}\right)\right)+20+e \end{array}$	30, 100, 500	[−32, 32]	0	Multimodal
Generalized Criewank Function	$f_{18}(x)=\frac{1}{4000}\sum_{i=1}^{n}x_{i}^{2}-\prod_{i=1}^{n}\cos\left(\frac{x_{i}}{\sqrt{i}}\right)+1$	30, 100, 500	[−600, 600]	0	Multimodal
Penalized 1	$\begin{array}{l} f_{19}\left(x\right)=\frac{\pi }{n}\left\{\begin{array}{l} 10\sin(\pi \mathrm{yi})+\sum_{i=1}^{n-1}{\left(y_{i}-1\right)}^{2}\left[1+10\sin^{2}(\pi y_{i+1})\right]\\ +{\left(y_{n}-1\right)}^{2} \end{array}\right\}+\\ \sum_{i=1}^{n}u(xi,10,100,4)\\ y_{i}=1+\frac{x_{i}+1}{4},u(xi,a,k,m)=\left\{\begin{array}{l} k{\left(x_{i}-a\right)}^{m},x_{i}>a\\ 0,-a<x_{i}<a\\ k{\left(-x_{i}-a\right)}^{m},x_{i}<-a \end{array}\right\} \end{array}$	30, 100, 500	[−50, 50]	0	Multimodal
Penalized 2	$\begin{array}{l} f_{20}(x)=0.1\left\{\begin{array}{l} \sin^{2}\left(3\pi x_{1}\right)+\sum_{i=1}^{n}{\left(x_{i}-1\right)}^{2}\left[1+\sin^{2}\left(3\pi x_{i}+1\right)\right]\\ +{\left(x_{n}-1\right)}^{2}\left[1+\sin^{2}\left(2\pi x_{n}\right)\right] \end{array}\right\}+\\ \sum_{i=1}^{n}u\left(x_{i},5,100,4\right) \end{array}$	30, 100, 500	[−50, 50]	0	Multimodal
Shekell’s Foxholes Function	$f_{21}(x)={\left(\frac{1}{500}+\sum_{j=1}^{25}\frac{1}{j+\sum_{i=1}^{2}{\left(x_{i}-a_{ij}\right)}^{6}}\right)}^{-1}$	2	[−65.536, 65.536]	1	Multimodal
Kowalik’s Function	$f_{22}(x)=\sum_{i=1}^{11}{\left[a_{i}-\frac{x_{1}\left(b_{i}^{2}+b_{i}x_{2}\right)}{b_{i}^{2}+b_{i}x_{3}+x_{4}}\right]}^{2}$	4	[−5, 5]	0.0003	Multimodal
Six-Hump Camel-Back Function	$f_{23}(x)=4x_{1}^{2}-2.1x_{1}^{4}+\frac{1}{3}x_{1}^{6}+x_{1}x_{2}-4x_{2}^{2}+4x_{2}^{4}$	2	[−5, 5]	−1.0316	Multimodal
Branin Function	$f_{24}(x)={\left(x_{2}-\frac{5.1}{4\pi^{2}}x_{1}^{2}+\frac{5}{\pi }x_{1}-6\right)}^{2}+10\left(1-\frac{1}{8\pi }\right)\cos x_{1}+10$	2	[−5, 5]	0.398	Multimodal
Goldstein-Price Function	$\begin{array}{l} f_{25}(x)=\left[1+{\left(x_{1}+x_{2}+1\right)}^{2}\left(19-14x_{1}+3x_{1}^{2}-14x_{2}+6x_{1}x_{2}+3x_{2}^{2}\right)\right]\\ \times \left[30+{\left(2x_{1}-3x_{2}\right)}^{2}\times \left(18-32x_{1}+12x_{1}^{2}+48x_{2}-36x_{1}x_{2}+27x_{2}^{2}\right)\right] \end{array}$	2	[−2, 2]	3	Multimodal
Hatman’s Function1	$f_{26}(x)=-\sum_{i=1}^{4}c_{i}\exp\left(-\sum_{j=1}^{3}a_{ij}{\left(x_{j}-p_{ij}\right)}^{2}\right)$	3	[0, 1]	−3.86	Multimodal
Hatman’s Function2	$f_{27}(x)=-\sum_{i=1}^{4}c_{i}\exp\left(-\sum_{j=1}^{6}a_{ij}{\left(x_{j}-p_{ij}\right)}^{2}\right)$	6	[0, 1]	−3.32	Multimodal
Schekel’s Family 1	$f_{28}(x)=-\sum_{i=1}^{5}{\left[\left(X-a_{i}\right){\left(X-a_{i}\right)}^{T}+c_{i}\right]}^{-1}$	4	[0, 10]	−10.1532	Multimodal
Schekel’s Family 2	$f_{29}(x)=-\sum_{i=1}^{7}{\left[\left(X-a_{i}\right){\left(X-a_{i}\right)}^{T}+c_{i}\right]}^{-1}$	4	[0, 10]	−10.4028	Multimodal
Schekel’s Family 3	$f_{30}(x)=-\sum_{i=1}^{10}{\left[\left(X-a_{i}\right){\left(X-a_{i}\right)}^{T}+c_{i}\right]}^{-1}$	4	[0, 10]	−10.5364	Multimodal

## References

[1] Yuan Y., Wei J., Huang H., Jiao W., Wang J., Chen H. (2023). Review of resampling techniques for the treatment of imbalanced industrial data classification in equipment condition monitoring. Eng. Appl. Artif. Intell..

[2] Liang Y.-C., Minanda V., Gunawan A. (2022). Waste collection routing problem: A mini-review of recent heuristic approaches and applications. Waste Manag. Res..

[3] Kuo R., Li S.-S. (2023). Applying particle swarm optimization algorithm-based collaborative filtering recommender system considering rating and review. Appl. Soft Comput..

[4] Fan S.-K.S., Lin W.-K., Jen C.-H. (2022). Data-driven optimization of accessory combinations for final testing processes in semiconductor manufacturing. J. Manuf. Syst..

[5] Huynh N.-T., Nguyen T.V.T., Tam N.T.T., Nguyen Q. (2021). Optimizing Magnification Ratio for the Flexible Hinge Displacement Amplifier Mechanism Design. Lecture Notes in Mechanical Engineering, Proceedings of the 2nd Annual International Conference on Material, Machines and Methods for Sustainable Development (MMMS2020), Nha Trang, Vietnam, 12–15 November 2020.

[6] Kler R., Gangurde R., Elmirzaev S., Hossain M.S., Vo N.T.M., Nguyen T.V.T., Kumar P.N. (2022). Optimization of Meat and Poultry Farm Inventory Stock Using Data Analytics for Green Supply Chain Network. Discret. Dyn. Nat. Soc..

[7] Yu K., Liang J.J., Qu B., Luo Y., Yue C. (2021). Dynamic Selection Preference-Assisted Constrained Multiobjective Differential Evolution. IEEE Trans. Syst. Man Cybern. Syst..

[8] Yu K., Sun S., Liang J.J., Chen K., Qu B., Yue C., Wang L. (2023). A bidirectional dynamic grouping multi-objective evolutionary algorithm for feature selection on high-dimensional classification. Inf. Sci..

[9] Tang J., Liu G., Pan Q. (2021). A review on representative swarm intelligence algorithms for solving optimization problems: Applications and trends. IEEE/CAA J. Autom. Sin..

[10] Yu K., Zhang D., Liang J.J., Chen K., Yue C., Qiao K., Wang L. (2023). A Correlation-Guided Layered Prediction Approach for Evolutionary Dynamic Multiobjective Optimization. IEEE Trans. Evol. Comput..

[11] Wei J., Huang H., Yao L., Hu Y., Fan Q., Huang D. (2021). New imbalanced bearing fault diagnosis method based on Sample-characteristic Oversampling TechniquE (SCOTE) and multi-class LS-SVM. Appl. Soft Comput..

[12] Yu K., Zhang D., Liang J.J., Qu B., Liu M., Chen K., Yue C., Wang L. (2023). A Framework Based on Historical Evolution Learning for Dynamic Multiobjective Optimization. IEEE Trans. Evol. Comput..

[13] Seyyedabbasi A., Kiani F. (2023). Sand Cat swarm optimization: A nature-inspired algorithm to solve global optimization problems. Eng. Comput..

[14] Chu S.-C., Tsai P.-W., Pan J.-S. (2006). Cat swarm optimization. PRICAI 2006: Trends in Artificial Intelligence, Proceedings of the 9th Pacific Rim International Conference on Artificial Intelligence, Guilin, China, 7–11 August 2006.

[15] Mirjalili S., Mirjalili S.M., Lewis A. (2014). Grey wolf optimizer. Adv. Eng. Softw..

[16] Mirjalili S., Lewis A. (2016). The whale optimization algorithm. Adv. Eng. Softw..

[17] Mirjalili S., Gandomi A.H., Mirjalili S.Z., Saremi S., Faris H., Mirjalili S.M. (2017). Salp Swarm Algorithm: A bio-inspired optimizer for engineering design problems. Adv. Eng. Softw..

[18] Rashedi E., Nezamabadi-Pour H., Saryazdi S. (2009). GSA: A gravitational search algorithm. Inf. Sci..

[19] Kennedy J., Eberhart R. Particle swarm optimization. Proceedings of the ICNN’95—International Conference on Neural Networks.

[20] Hayyolalam V., Kazem A.A.P. (2020). Black widow optimization algorithm: A novel meta-heuristic approach for solving engineering optimization problems. Eng. Appl. Artif. Intell..

[21] Hu G., Guo Y., Wei G., Abualigah L. (2023). Genghis Khan shark optimizer: A novel nature-inspired algorithm for engineering optimization. Adv. Eng. Inform..

[22] Heidari A.A., Mirjalili S., Faris H., Aljarah I., Mafarja M., Chen H. (2019). Harris hawks optimization: Algorithm and applications. Future Gener. Comput. Syst..

[23] Hashim F.A., Hussien A.G. (2022). Snake Optimizer: A novel meta-heuristic optimization algorithm. Knowl.-Based Syst..

[24] Xue J., Shen B. (2023). Dung beetle optimizer: A new meta-heuristic algorithm for global optimization. J. Supercomput..

[25] Jia H., Rao H., Wen C., Mirjalili S. (2023). Crayfish optimization algorithm. Artif. Intell. Rev..

[26] Wei J., Wang J., Huang H., Jiao W., Yuan Y., Chen H., Wu R., Yi J. (2023). Novel extended NI-MWMOTE-based fault diagnosis method for data-limited and noise-imbalanced scenarios. Expert Syst. Appl..

[27] Kiani F., Nematzadeh S., Anka F.A., Fındıklı M. (2023). Chaotic Sand Cat Swarm Optimization. Mathematics.

[28] Seyyedabbasi A. (2023). Binary Sand Cat Swarm Optimization Algorithm for Wrapper Feature Selection on Biological Data. Biomimetics.

[29] Qtaish A., Albashish D., Braik M., Alshammari M.T., Alreshidi A., Alreshidi E. (2023). Memory-Based Sand Cat Swarm Optimization for Feature Selection in Medical Diagnosis. Electronics.

[30] Wu D., Rao H., Wen C., Jia H., Liu Q., Abualigah L. (2022). Modified Sand Cat Swarm Optimization Algorithm for Solving Constrained Engineering Optimization Problems. Mathematics.

[31] Kiani F., Anka F.A., Erenel F. (2023). PSCSO: Enhanced sand cat swarm optimization inspired by the political system to solve complex problems. Adv. Eng. Softw..

[32] Yao L., Yuan P., Tsai C.-Y., Zhang T., Lu Y., Ding S. (2023). ESO: An Enhanced Snake Optimizer for Real-world Engineering Problems. Expert Syst. Appl..

[33] Yuan P., Zhang T., Yao L., Lu Y., Zhuang W. (2022). A Hybrid Golden Jackal Optimization and Golden Sine Algorithm with Dynamic Lens-Imaging Learning for Global Optimization Problems. Appl. Sci..

[34] Yao L., Li G., Yuan P., Yang J., Tian D., Zhang T. (2023). Reptile Search Algorithm Considering Different Flight Heights to Solve Engineering Optimization Design Problems. Biomimetics.

[35] Zhong C., Li G., Meng Z. (2022). Beluga whale optimization: A novel nature-inspired metaheuristic algorithm. Knowl.-Based Syst..

[36] Abed-Alguni B.H., Alawad N.A., Al-Betar M.A., Paul D. (2023). Opposition-based sine cosine optimizer utilizing refraction learning and variable neighborhood search for feature selection. Appl. Intell..

[37] Ma C., Huang H., Fan Q., Wei J., Du Y., Gao W. (2022). Grey wolf optimizer based on Aquila exploration method. Expert Syst. Appl..

[38] Wu R., Huang H., Wei J., Ma C., Zhu Y., Chen Y., Fan Q. (2022). An improved sparrow search algorithm based on quantum computations and multi-strategy enhancement. Expert Syst. Appl..

[39] Fan Q., Huang H., Chen Q., Yao L., Yang K., Huang D. (2021). A modified self-adaptive marine predators algorithm: Framework and engineering applications. Eng. Comput..

[40] Wang Y., Ran S., Wang G.-G. Role-Oriented Binary Grey Wolf Optimizer Using Foraging-Following and Lévy Flight for Feature Selection. Appl. Math. Model..

[41] Lahmar I., Zaier A., Yahia M., Boaullègue R. (2023). A Novel Improved Binary Harris Hawks Optimization For High dimensionality Feature Selection. Pattern Recognit. Lett..

[42] Turkoglu B., Uymaz S.A., Kaya E. (2022). Binary Artificial Algae Algorithm for feature selection. Appl. Soft Comput..

[43] Hu G., Zhong J., Zhao C., Wei G., Chang C.-T. (2023). LCAHA: A hybrid artificial hummingbird algorithm with multi-strategy for engineering applications. Comput. Methods Appl. Mech. Eng..

[44] Long W., Jiao J., Xu M., Tang M., Wu T., Cai S. (2022). Lens-imaging learning Harris hawks optimizer for global optimization and its application to feature selection. Expert Syst. Appl..

[45] Chen H., Xu Y., Wang M., Zhao X. (2019). A balanced whale optimization algorithm for constrained engineering design problems. Appl. Math. Model..

[46] Jia H., Li Y., Wu D., Rao H., Wen C., Abualigah L. (2023). Multi-strategy Remora Optimization Algorithm for Solving Multi-extremum Problems. J. Comput. Des. Eng..

[47] Nadimi-Shahraki M.H., Zamani H. (2022). DMDE: Diversity-maintained multi-trial vector differential evolution algorithm for non-decomposition large-scale global optimization. Expert Syst. Appl..

[48] Holland J.H. (1992). Genetic algorithms. Sci. Am..

[49] Dorigo M., Birattari M., Stutzle T. (2006). Ant colony optimization. IEEE Comput. Intell. Mag..

[50] Dhargupta S., Ghosh M., Mirjalili S., Sarkar R. (2020). Selective opposition based grey wolf optimization. Expert Syst. Appl..

[51] Komathi C., Umamaheswari M. (2019). Design of gray wolf optimizer algorithm-based fractional order PI controller for power factor correction in SMPS applications. IEEE Trans. Power Electron..

[52] Ziyu T., Dingxue Z. A modified particle swarm optimization with an adaptive acceleration coefficients. Proceedings of the 2009 Asia-Pacific Conference on Information Processing.

[53] Ewees A.A., Ismail F.H., Sahlol A.T. (2023). Gradient-based optimizer improved by Slime Mould Algorithm for global optimization and feature selection for diverse computation problems. Expert Syst. Appl..

[54] Sun L., Si S., Zhao J., Xu J., Lin Y., Lv Z. (2023). Feature selection using binary monarch butterfly optimization. Appl. Intell..

